# Supplementary material for: Camrelizumab and apatinib plus induction chemotherapy and concurrent chemoradiotherapy in stage N3 nasopharyngeal carcinoma: a phase 2 clinical trial
Source: Nat Commun. 2024 Feb 3;15:1029. doi: 10.1038/s41467-024-45126-0 (PMC10838332; doi:10.1038/s41467-024-45126-0)
Supplement: Supplementary file 1 — Supplementary Information [file 41467_2024_45126_MOESM1_ESM.pdf]

Supplementary information

Contents

Supplementary Methods....................3
Dose modifications and toxicity prevention.................... 3
Description of the guidelines for intensity-modulated radiation therapy in this trial........... 5
Definitions of the endpoints 8
Per-protocol analysis 9
Supplementary Tables....................10
Supplementary Table 2. Patient baseline characteristics of per-protocol population.......... 10
Supplementary Table 3. Survival analysis in per-protocol population.................... 11
Supplementary Table 4. Characteristics of the patients at baseline between QUINTUPLED and historical cohorts.................... 12
Supplementary Table 5. Compliance to induction chemotherapy.................... 13
Supplementary Table 6. Compliance to camrelizumab and apatinib. 14
Supplementary Table 7. Compliance to concurrent cisplatin chemotherapy and radiotherapy.................... 15
Supplementary Table 8. Characteristics between patients received or refused to biopsy... 16
Supplementary Table 9. Treatment-related adverse events, according to treatment phase and grade. 17
Supplementary Table 10. Immune-related adverse events, according to treatment phase and grade..................... 19
Supplementary Figures....................20
Supplementary Figure 1. Response to induction therapy.................... 20
Supplementary Figure 2. Representative MRI comparison of pre- and after induction phase. 21
Supplementary Figure 3. Pathology of nasopharyngeal and lymph node tissues............... 22
Supplementary Figure 4. Trajectories of plasma EBV DNA levels.................... 23
Supplementary Figure 5. Immune-related toxic epidermal necrolysis-like skin reaction... 24
Supplementary Figure 6. Kaplan-Meier analysis of distant metastasis-free survival, failure-free survival, overall survival, and locoregional recurrence-free survival (intention-to-treat population).................... 25
Supplementary References....................26
Supplementary Note 1. Study Protocol....................27

Supplementary Methods

Dose modifications and toxicity prevention

Patients were examined and graded for subjective or objective evidence of toxic effects of treatment according to the National Cancer Institute Common Terminology Criteria for Adverse Events (CTCAE; version 5) every week when the induction and concurrent chemotherapy and radiotherapy was administered and before each camrelizumab dose. Radiotherapy dose modifications were not allowed.

Chemotherapy dose adjustments were allowed in cases of hematological or non-hematological toxicity. In the case of hematological toxicity, during the induction and concurrent phase, chemotherapy was withheld until the nadir values were 1500 cells per μL or higher for neutrophils and 100,000 cells per μL or higher for platelets. In the case of renal or liver toxicity, chemotherapy was withheld until adequate renal and liver functions were regained.

Dose reductions of camrelizumab were not allowed. Dose interruptions and dose reductions (maximum of two reductions) of apatinib were permitted for toxicities that were not relieved by supportive care. The first dose reduction was to 250 mg once per day with 5 days on and 2 days off, and additional reduction was to 250 mg once per day every other day. If the apatinib dose was reduced, it could not be increased later. In the middle of the study, the initial dose of apatinib was adjusted with 250 mg once per day with 5 days on and 2 days off.

To prevent chemotherapy-induced nausea and vomiting, we recommended 5-HT3-receptor antagonist (ondansetrone 8 mg or granisetron 3 mg, intravenously) and NK-1-receptor antagonist (aprepitant 125 mg on day 1 and 80 mg on days 2 and 3); metoclopramide (10 mg intramuscularly) could also be used if symptoms persisted despite the triplet combination. To prevent cisplatin-induced nephrotoxicity, we administered a 3-day hydration protocol on days 0–2 and used diuretics (mannitol 50 g intravenously and furosemide 20 mg intravenously) on the day of cisplatin administration. Severe malnutrition or continuous weight loss during treatment was an indication for parenteral or nasogastric nutritional supplementation. All of the supportive care could be delivered in both inpatient and outpatient settings at the physician’s discretion. Polyethylene glycol-conjugated recombinant human granulocyte stimulating factor is an alternative agent for the prevention of serious neutropenia. For grade 4 neutropenia, prophylactic antibiotics were

administered.

Description of the guidelines for intensity-modulated radiation therapy in this trial

In general, within 1-2 weeks after the third course of induction chemotherapy (IC), all patients were immobilized in the supine position using a thermoplastic mask that covered the head, neck, and shoulder. Both non-enhanced CT (for dose calculation) and contrast-enhanced CT (for target delineation) images were obtained from the vertex to 2 cm below the sternoclavicular joint, with 3-mm slices. MRI fusion with simulation CT images were performed to assist the targets delineation. Target volumes were defined in accordance with the International Commission on Radiation Units and Measurements (ICRU) reports. Gross tumor volume (GTV) included the primary tumor (GTVnx) and enlarged lymph nodes (LN, GTVnd). The primary tumor volumes were defined as all the known gross disease of soft tissues after IC and areas of violated bone before treatment. The LN volumes was defined as all the known gross disease after IC. High-risk clinical target volume ($CTV_{high-risk}$) was defined as the nasopharyngeal GTV plus a 5-10 mm margin (2-3 mm posteriorly if adjacent to the brainstem or spinal cord) to encompass the high-risk sites of microscopic extension and the whole nasopharynx. Low-risk clinical target volume ($CTV_{low-risk}$) was defined as the high-risk clinical target volume plus a 5-10 mm margin (2-3 mm posteriorly if adjacent to the brainstem or spinal cord) to encompass the low-risk sites of microscopic extension, including the skull base, clivus, sphenoid sinus, parapharyngeal space, pterygoid fossa, retropharyngeal nodal regions, and elective neck area from level II to level V. Area Ib mainly needs preventive irradiation in the following high-risk groups: submandibular gland involvement, disease involving the anatomical structure of the lymph node drainage area (oral and nasal front half) with area Ib as the first stop, lymph node invasion in area II accompanied by extracapsular invasion, or in cases where the maximum diameter of the lymph node in area II exceeds 2 cm. For the planning target volumes of GTVnx, GTVnd, $CTV_{high-risk}$, and $CTV_{low-risk}$, total radiation doses of 68–72 Gy, 64–68 Gy, 60-64 Gy, and 54-58 Gy, respectively, were administered in 30-33 fractions, delivered daily at five fractions per week, starting on the first day of the first concurrent chemoradiotherapy cycle.

The normal tissue dose constraints are listed in Table SS1. All plans were generated by a team of dosimetrists using a wholefield (including neck radiation) simultaneous integrated boost technique. In general, when critical normal tissues (e.g., brain stem and spinal cord) were adjacent to the high-dose target volumes, the target volume coverage could be compromised to keep these critical normal

tissues within the dose constraints. When other normal tissues of lower priority were adjacent to the high-dose target volumes, the dose to these tissues was kept as low as possible without compromising the target coverage. The trade-off between covering the target volume and protecting normal tissue in each case was discussed and decided upon by the research team at each institution. Radiation was delivered once daily at five fractions per week. It was recommended that patients commence chemoradiotherapy within 21-28 days from the first day of the last cycle of induction chemotherapy.

Supplementary Table 1. Normal tissue dose constraint structure.

Structure	Dose constraints
**Critical organ at risk**	
Brain stem	Dmax ≤ 54 Gy
PRV-Brain stem	D1 ≤ 60 Gy
Spinal cord Dmax*	Dmax* ≤ 45 Gy
PRV-Spinal cord	D1†≤ 50 Gy
Optic nerve	Dmax ≤ 54 Gy
PRV-Optic nerve	D1 ≤ 60 Gy
Optic chiasm	Dmax ≤ 54 Gy
PRV-Optic chiasm	D1 ≤ 60 Gy
Temporal lobe	Dmax ≤ 60 Gy
PRV-Temporal lobe	D1 ≤ 65 Gy
**Intermediate-risk organ at risk**	
Pituitary	Dmax < 60 Gy
Mandibular	Dmax < 70 Gy
Temporomandibular Joint	Dmax < 70 Gy
Lens	Dmean‡ < 8 Gy
Eyes	Dmean < 35 Gy
**Low-risk organ at risk**	
Parotid	Dmean < 26 Gy
Parotid	V30¶ < 50%
Cochlea	Dmean < 50 Gy
Tongue	Dmean < 45 Gy
Larynx	Dmean < 45 Gy

PRV = planning organ at risk volume.
* Maximum point dose to the target volume.
† Dose received by 1% of the target volume.
‡ Mean dose to the target volume.
¶ At least 50% of the gland will receive <30 Gy (should be achieved in at least one gland)

Plasma Epstein-Barr virus (EBV) DNA extraction and real-time quantitative polymerase chain reaction analysis.

Peripheral blood (3 ml) was obtained in an EDTA tube and centrifuged at 1600 x g for 15 min for isolation of plasma and peripheral blood mononuclear cells. Then Viral DNA was extracted using the QIAamp Blood Kit (Qiagen, Hilden, Germany) and stored at -80°C until further processing. A total of 500 μl plasma samples were used for DNA extraction per column and a final elution volume of 50 μl was used to elute the DNA from the extraction column. A real-time quantitative polymerase chain reaction (PCR) system was developed for plasma EBV DNA detection toward the BamHI-W region of the EBV genome. The sequences consisted of the amplification primers W-44F (5'-AGT CTC TGC CTC AGG GCA-3') and W-119R (5'-ACA GAG GGC CTG TCC ACCG-3') and the dual-labeled fluorescent probe W-67T (5'-[FAM] CAC TGT CTG TAA AGT CCA GCC TCC[TAMRA]-3'). Quantification testing of plasma Epstein-Barr virus (EBV) DNA levels were based on a lower limit of detection cutoff value of 1000 copies/mL. Persistently negative EBV DNA level is defined as a quantitative EBV DNA level reported as 0 copies/mL and also reported as zero on subsequent tests.

Definitions of the endpoints

Distant metastasis-free survival was calculated from the date of treatment initiation to the date of documented distant metastasis; overall survival was calculated from the date of treatment initiation to death; failure-free survival was calculated from the date of treatment initiation to the date of documented distant metastasis, locoregional recurrence, or death from any cause, whichever occurred first; locoregional recurrence-free survival was calculated from the date of treatment initiation to the date of documented locoregional recurrence. Patients with a distant metastasis failure as a first event were censored for locoregional recurrence and vice versa. If both distant metastasis and locoregional recurrences occurred at the same time, patients were considered as having an event for both distant metastasis-free survival and locoregional recurrence-free survival. Patients who were lost to follow-up, or were still alive without distant metastasis or locoregional recurrence were censored at the date of last follow-up. Treatment responses were categorized as complete response, partial response, stable disease and progressive disease based on the Response Evaluation Criteria in Solid Tumors (version 1.1).[1]

Per-protocol analysis

The per-protocol population comprised of cases that completed radical radiotherapy and at least one dose of induction therapy. Supplementary Table 2 lists baseline characteristics of per-protocol population. The efficacy in per-protocol population is summarized in the Supplementary Table 3.

Supplementary Tables

Supplementary Table 2. Patient baseline characteristics of per-protocol population.

Characteristic	No. (%) of patients (N=47)
Age, year	
Median (interquartile range)	45 (37-51)
Range	20-65
Sex	
Male	35 (74.5)
Female	12 (25.5)
ECOG perform status*	
0	37 (78.7)
1	10 (21.3)
Tumor category†	
T1	3 (6.4)
T2	7 (14.9)
T3	26 (55.3)
T4	11 (23.4)
Histology classification	
Non-keratinising differentiated	1 (2.1)
Non-keratinising undifferentiated	46 (97.9)
Plasma EBV DNA level, median (interquartile range), copies/mL‡	4710 (1680-17200)

* Eastern Cooperative Oncology Group (ECOG) performance-status scores range from 0 to 5, with higher scores indicating greater disability.

† Tumor category refer to the T stage according to the TNM staging system (American Joint Committee on Cancer, 8th edition).

‡ Shown are the data of pretreatment plasma Epstein-Barr virus (EBV) DNA levels which were based on a detectable cutoff value of 1000 copies/mL.

Supplementary Table 3. Survival analysis in per-protocol population.

	Evaluable patients (n=47)
Distant metastasis-free survival	
Distant failures	1 (2.1%)
Proportion of patients without distant failures at 2 year (95% CI)	97.9% (85.7-99.7)
Locoregional failure-free survival	
Locoregional failures	1 (2.1%)
Proportion of patients without distant failures at 2 year (95% CI)	97.8% (86.3-99.7)
Overall survival	
Death	1 (2.1%)
Proportion of patients alive at 2 year (95% CI)	97.9% (85.7-99.7)
Failure-free survival	
Failures	2 (4.3%)
Proportion of failure-free patients at 2 year (95% CI)	95.7% (84.4-98.9)

IC = induction chemotherapy.

Supplementary Table 4. Characteristics of the patients at baseline between QUINTUPLED and historical cohorts.

Characteristic	Patients, No. (%)		P value
	QUINTUPLED cohort (N=49)	Historical cohort[2] (N=111)	
Age, year			0.64
Median (interquartile range)	46 (37-52)	46 (38-51)	
Range	20-65	18-65	
Sex			0.60
Male	37 (75.5)	88 (79.3)	
Female	12 (24.5)	23 (20.7)	
ECOG perform status†			0.10
0	39 (79.6)	99 (89.2)	
1	10 (20.4)	12 (10.8)	
Tumor category‡			0.39
T1	3 (6.1)	5 (4.5)	
T2	7 (14.3)	7 (6.3)	
T3	26 (53.1)	66 (59.5)	
T4	13 (26.5)	33 (29.7)	
Histology classification			1.00
Non-keratinising differentiated	1 (2.0)	2 (1.8)	
Non-keratinising undifferentiated	48 (98.0)	109 (98.2)	
Plasma EBV DNA level, median (interquartile range), copies/mL§	4710 (1670-17900)	3670 (1150-27400)¶	0.057

P values were calculated by two-sided chi-square test (or Fisher exact test) or the t test.

† Eastern Cooperative Oncology Group (ECOG) performance-status scores range from 0 to 5, with higher scores indicating greater disability.

‡ Tumor category refer to the T stage according to the TNM staging system (American Joint Committee on Cancer, 8th edition).

§ Shown are the data of pretreatment plasma Epstein-Barr virus (EBV) DNA level which were based on a lower limit of detection cutoff value of 1000 copies/mL.

¶ Two patients' pretreatment plasma EBV DNA level were unmeasured in historical cohort.

Supplementary Table 5. Compliance to induction chemotherapy.

Variable	No. (%) of patients
No. patients evaluated	49
Patients completing at least two cycle IC, no. (%)	49 (100%)
Patients completing three cycle IC, no. (%)	49 (100%)
Patients with dose reductions	5 (10.2)
Dose reductions of NAB-paclitaxel	1 (2%)
Dose reductions of cisplatin	3 (6%)
Dose reductions of capecitabine	4 (8%)
Reason for dose modification, no. (%)	
Hand-foot syndrome	2 (4.1)
Gastrointestinal	2 (4.1)
Thrombocytopenia	1 (2.0)
Patients with at least one cycle delay > 3 days, no. (%)	16 (33%)
Patients with at least one cycle delay > 7 days, no. (%)	8 (16%)
Reason for delay, no. (%)	
Treatment-related adverse event	5 (10%)
Other*	11 (22%)
Median (IQR) duration of IC (days)	44 (42-49)
Median (IQR) interval between last day of IC and first day of RT (days)	19 (16-23)
Median (IQR) interval between first day of IC to first day of RT (days)	68 (64-72)

IC = induction chemotherapy; IQR = interquartile range; RT = radiotherapy.

* Other reasons for treatment delays included logistics, personal reasons, or other non-treatment relative factors, i.e., COVID-19 epidemic prevention travel policy.

Supplementary Table 6. Compliance to camrelizumab and apatinib.

Variable	No. (%) of patients
No. patients evaluated	49
Patients receiving camrelizumab, no. (%)	49 (100)
Median (interquartile range) cycles of camrelizumab	12 (9-16)
Patients with dose interruptions	22 (44.9)
Reason for dose interruptions, no. (%)	
Disease recurrence	2 (4.1)
Reactive capillary endothelial proliferation	3 (6.1)
Abnormal inflammatory cytokines	3 (6.1)
Plasma cortisol abnormalities	3 (6.1)
Hypothyroidism	2 (4.1)
Rash/epidermal necrolysis-like skin reaction	2 (4.1)
Thrombocytopenia	1 (2.0)
Anemia	1 (2.0)
Other reasons*	5 (10.2)
Patients receiving apatinib, no. (%)	49 (100)
Median (interquartile range) dose of apatinib (mg)	7500 (5000-10125†)
Patients receiving at least 3750 mg (one cycle dose)	44 (89.8)
Patients receiving at least 7500 mg (two cycle doses)	34 (69.4)
Patients receiving at least 10000 mg (three cycle doses) †	21 (42.9)
Patients with dose interruptions of apatinib	26 (53.1)
Reason for dose interruptions, no. (%)	
Rash	10 (20.6)
Hand-foot syndrome	2 (4.1)
Nasal bleeding	2 (4.1)
Dermatitis	2 (4.1)
Pruritus	2 (4.1)
Fatigue	1 (2.0)
Diarrhea	1 (2.0)
Vomit	1 (2.0)
Increased blood pressure	1 (2.0)
Thrombocytopenia	1 (2.0)
Other reasons*	3 (6.1)
Patients with dose reduction of apatinib	3 (6.1)
Reason for dose reduction, no. (%)	
Rash	2 (4.1)
Hand-foot syndrome	1 (2.0)

* Other reasons for treatment delays included logistics, personal reasons, or other non-treatment relative factors, i.e., COVID-19 epidemic prevention travel policy.

† At the beginning of the study, the initial dose of apatinib was 250 mg daily from day 1 to day 56; from patient #26, with the protocol modification, the initial dose of apatinib adjusted with 250 mg once per day with 5 days on and 2 days off in second (final) version protocol for total 8 weeks (It was recommended that patients stopped apatinib at least 7 days before starting IMRT).

Supplementary Table 7. Compliance to concurrent cisplatin chemotherapy and radiotherapy.

Variable	No. (%) of patients
No. patients evaluated	49
Patients receiving RT, no. (%)	48
Patients completing RT, no. (%)	47 (98%)*
Median (IQR) dose of RT (Gy)	6930 (6900-6990)
Median (IQR) fraction	30 (30-32)
Median (IQR) dose per fraction (Gy)	2.30 (2.16-2.33)
Median (IQR) duration of RT (days)	44 (42-46)
Patients starting concurrent cisplatin, no. (%)	48
Patients completing at least one cycles CC, no. (%)	48 (100%)
Patients completing two cycles CC, no. (%)	41 (85%)
Reason for discontinuation, no. (%)	
Refused	4 (8.3)
Non-hematological	3 (6.3)
Patients with dose reductions of concurrent cisplatin, no. (%)	2 (4%)
Reason for dose reductions, no. (%)	
Both hematological and non-hematological	1 (2.1)
Non-hematological	1 (2.1)

CC = concurrent chemotherapy; IQR = interquartile range; RT = radiotherapy.

* One patient whose radiotherapy was interrupted prematurely after receiving 9 fractions because of personal reason.

Supplementary Table 8. Characteristics of patients who received or refused biopsy.

Characteristic	Endoscopic biopsy		Lymph nodes biopsy	
	Received (N=33)	Refused (N=16)	Received (N=34)	Refused (N=15)
Age, year				
Median (IQR)	45 (38-50)	49 (36-58)	43 (37-50)	48 (40-56)
Range	20-65	34-65	20-65	34-65
Gender				
Male	25 (75.8)	12 (75.0)	25 (73.5)	12 (80.0)
Female	8 (24.2)	4 (25.0)	9 (26.5)	3 (20.0)
ECOG perform status*				
0	28 (84.8)	11 (68.8)	28 (84.8)	11 (73.3)
1	5 (15.2)	5 (31.3)	6 (17.6)	4 (26.7)
Tumor category†				
T1	2 (6.1)	1 (6.3)	1 (2.9)	2 (13.3)
T2	4 (12.1)	3 (18.8)	4 (11.8)	3 (20.0)
T3	17 (51.5)	9 (56.3)	22 (64.7)	4 (26.7)
T4	10 (30.3)	3 (18.8)	7 (20.6)	6 (40.0)
Histology classification				
Non-keratinising differentiated	1 (3)	0 (0)	0 (0)	1 (6.7)
Non-keratinising undifferentiated	32 (97)	16 (100)	34 (100)	14 (93.3)
Plasma EBV DNA level, median (interquartile range), copies/mL‡	2970 (1275-11650)	8425 (2970-44700)	6300 (1560-19950)	3240 (1660-12500)

IQR = interquartile range.

* Eastern Cooperative Oncology Group (ECOG) performance-status scores range from 0 to 5, with higher scores indicating greater disability.

† Tumor category refers to the T stage according to the TNM staging system (American Joint Committee on Cancer, 8th edition).

‡ Shown are the data of pretreatment plasma Epstein-Barr virus (EBV) DNA levels which were based on a lower limit of detection cutoff value of 1000 copies/mL.

Supplementary Table 9. Treatment-related adverse events, according to treatment phase and grade.

Event	Cumulative treatment-related adverse events (n=49)		Induction phase (n=49)		Concurrent phase (n=48)	
	Grade 1 or 2	Grade 3 or 4	Grade 1 or 2	Grade 3 or 4	Grade 1 or 2	Grade 3 or 4
Any treatment-related adverse event*	17 (34.7)	32 (65.3)	26 (53.1)	23 (46.9)	27 (56.2)	21 (43.8)
Leukopenia	31 (63.3)	7 (14.3)	15 (30.6)	3 (6.1)	23 (47.9)	2 (4.2)
Neutropenia	36 (73.5)	8 (16.3)	25 (51.0)	7 (14.3)	34 (70.8)	1 (2.1)
Anemia	41 (83.7)	7 (14.3)	43 (87.8)	0	42 (87.5)	4 (8.3)
Thrombocytopenia	16 (32.7)	5 (10.2)	10 (20.4)	1 (2.0)	13 (27.1)	2 (4.2)
Hypokalemia	23 (46.9)	4 (8.2)	20 (40.8)	2 (4.1)	15 (31.3)	1 (2.1)
Hyponatremia	23 (46.9)	2 (4.1)	20 (40.8)	1 (2.0)	10 (20.8)	1 (2.1)
Hypomagnesemia	31 (63.3)	0	14 (28.6)	0	27 (56.3)	0
hypermagnesemia	15 (30.6)	0	15 (30.6)	0	0	0
Hypocalcemia	30 (61.3)	0	29 (59.2)	0	9 (18.8)	0
hypercalcinemia	10 (20.4)	0	1 (2.0)	0	4 (8.3)	0
Hypoalbuminemia	27 (55.1)	0	24 (49.0)	0	18 (37.5)	0
Alanine aminotransferase increased	28 (57.1)	0	25 (51.0)	0	5 (10.4)	0
Aspartate aminotransferase increased	36 (73.5)	1 (2.0)	34 (69.4)	0	8 (16.7)	0
Lactate dehydrogenase increased	40 (81.6)	0	40 (81.6)	0	9 (18.8)	0
Blood creatinine increased	9 (18.4)	0	5 (10.2)	0	7 (14.6)	0
Hyperuricaemia	28 (57.1)	0	22 (44.9)	0	17 (35.4)	0
Hypercholesterolemia	32 (65.3)	1 (2.0)	22 (44.9)	1 (2.0)	16 (33.3)	0
Rash	22 (44.9)	5 (10.2)	21 (42.9)	5 (10.2)	4 (8.3)	0
Reactive capillary endothelial proliferation	42 (85.7)	0	25 (51.0)	0	34 (70.8)	0
Fatigue	39 (79.6)	2 (4.1)	37 (75.5)	2 (4.1)	38 (79.2)	0
Decreased appetite	43 (87.8)	0	43 (87.8)	0	42 (87.5)	0
Dry mouth	40 (83.7)	7 (14.3)	0	0	35 (72.9)	5 (10.4)
Mucositis	32 (65.3)	14 (28.6)	5 (10.2)	0	31 (64.6)	14 (29.2)
Dermatitis	32 (65.3)	3 (6.1)	0	0	32 (66.7)	3 (6.3)
Weight loss	31 (63.3)	1 (2.0)	6 (12.2)	0	27 (56.3)	1 (2.1)
hypogeusia	41 (83.7)	-	5 (10.2)	-	39 (81.3)	-
Nausea	41 (83.7)	7 (14.3)	41 (83.7)	5 (10.2)	45 (93.8)	2 (4.2)
Vomit	32 (65.3)	6 (12.2)	26 (53.1)	3 (6.1)	34 (70.8)	3 (6.3)
Hypertension	4 (8.2)	1 (2.0)	4 (8.2)	1 (2.0)	0	0
Diarrhea	6 (12.2)	2 (4.1)	4 (8.2)	2 (4.1)	3 (6.3)	0

Constipation	21 (42.9)	0	14 (28.6)	0	18 (37.5)	0
Dizziness	11 (22.4)	0	9 (18.4)	0	4 (8.3)	0
Pruritus	22 (44.9)	3 (6.1)	21 (42.9)	3 (6.1)	8 (16.7)	0
Hiccup	11 (22.4)	0	8 (16.3)	0	6 (12.5)	0
Hoarseness	18 (36.7)	0	8 (16.3)	0	9 (18.8)	0
Fever	7 (14.3)	0	3 (6.1)	0	2 (4.2)	0
Peripheral neuropathy	40 (81.6)	1 (2.0)	39 (79.6)	1 (2.0)	22 (45.8)	0
Allergic reaction	3 (6.1)	0	3 (6.1)	0	0	0
Any late adverse events†	39 (79.6)	8 (16.3)	-	-	-	-
Hearing impairment	20 (40.8)	5 (10.2)	-	-	-	-
Dry mouth	40 (81.6)	4 (8.2)	-	-	-	-
Trismus	4 (8.2)	0	-	-	-	-
Temporal lobe injury	6 (12.2)	0	-	-	-	-
Eye damage	3 (6.1)	0	-	-	-	-
Peripheral neuropathy	4 (8.2)	0	-	-	-	-

* Listed are all the treatment-related adverse events that occurred during the treatment period. The severity of treatment-related adverse events was graded according to the Common Terminology Criteria for Adverse Events, version 5.0, of the National Cancer Institute. Data are for grade 1-2 treatment-related adverse events occurred in at least 5% of the patients and all grade 3 and grade 4 adverse events. Patients may have had more than one event. No grade 5 treatment-related adverse events occurred.

† Late adverse events that occurred 12 weeks after the end of radiation therapy. Shown are late adverse events of interest that occurred in at least 5% of the patients and all grade 3 or 4 late adverse events. The severity of late adverse events was graded according to the Radiation Therapy Oncology Group and European Organization for Research and Treatment of Cancer late radiation morbidity scoring scheme.

Supplementary Table 10. Immune-related adverse events, according to treatment phase and grade.*

Event	Cumulative immune-related adverse events (n=49)		Induction phase (n=49)		Concurrent phase (n=48)		Maintain phase (n=48)	
	Grade 1 or 2	Grade 3 or 4	Grade 1 or 2	Grade 3 or 4	Grade 1 or 2	Grade 3 or 4	Grade 1 or 2	Grade 3 or 4
Any immune-related adverse events†	43 (87.8)	5 (10.2)	29 (59.2)	5 (10.2)	39 (81.3)	0	43 (87.8)	0
Reactive capillary endothelial proliferation‡	42 (85.7)	0	25 (51.0)	0	34 (70.8)	0	40 (81.6)	0
Hypothyroidism	15 (30.6)	0	7 (14.3)	0	3 (6.3)	0	14 (28.6)	0
Rash	5 (10.2)	3 (6.1)	4 (8.2)	3 (6.1)	1 (2.1)	0	0	0
Anti-thyroid antibody positive	13 (26.5)	0	8 (16.3)	0	7 (14.3)	0	12 (24.5)	0
Thyroglobulin decreased	15 (30.6)	0	11 (22.5)	0	9 (18.4)	0	10 (20.4)	0
Diarrhea	0	1 (2.0)	0	1 (2.0)	0	0	0	0
Brain natriuretic peptide increased	3 (6.1)	0	2 (4.1)	0	2 (4.1)	0	0	0
Lipase increased	5 (10.2)	0	3 (6.1)	0	0	0	2 (4.1)	0
Inflammatory cytokine increased	0	1 (2.0)	0	1 (2.0)	0	0	0	0

* Due to one patient who declined concurrent chemoradiotherapy after induction phase, there were 48 patients for safety analysis in concurrent and maintenance phase.

† Immune-related adverse events that occurred during the treatment period or within 30 days after the treatment period. The severity of immune--related adverse events was graded according to the Common Terminology Criteria for Adverse Events, version 5.0, of the National Cancer Institute. Shown are adverse events of interest that occurred in at least 3 patients or grade 3 or grade 4 adverse events. Patients may have had more than one event. No grade 5 immune-related adverse events occurred.

‡ Two cases have the reactive capillary endothelial proliferation at nasal, and nasopharynx mucosa, respectively.

Supplementary Figures

Supplementary Figure 1. Response to induction therapy. One week after completing induction therapy, the responses to induction therapy were assessed by imaging by independent image committee and were classified according to the Response Evaluation Criteria in Solid Tumors (RECIST) criteria, version 1.1. All patients (n=49) achieved objective response (complete and partial response) after completing induction therapy.

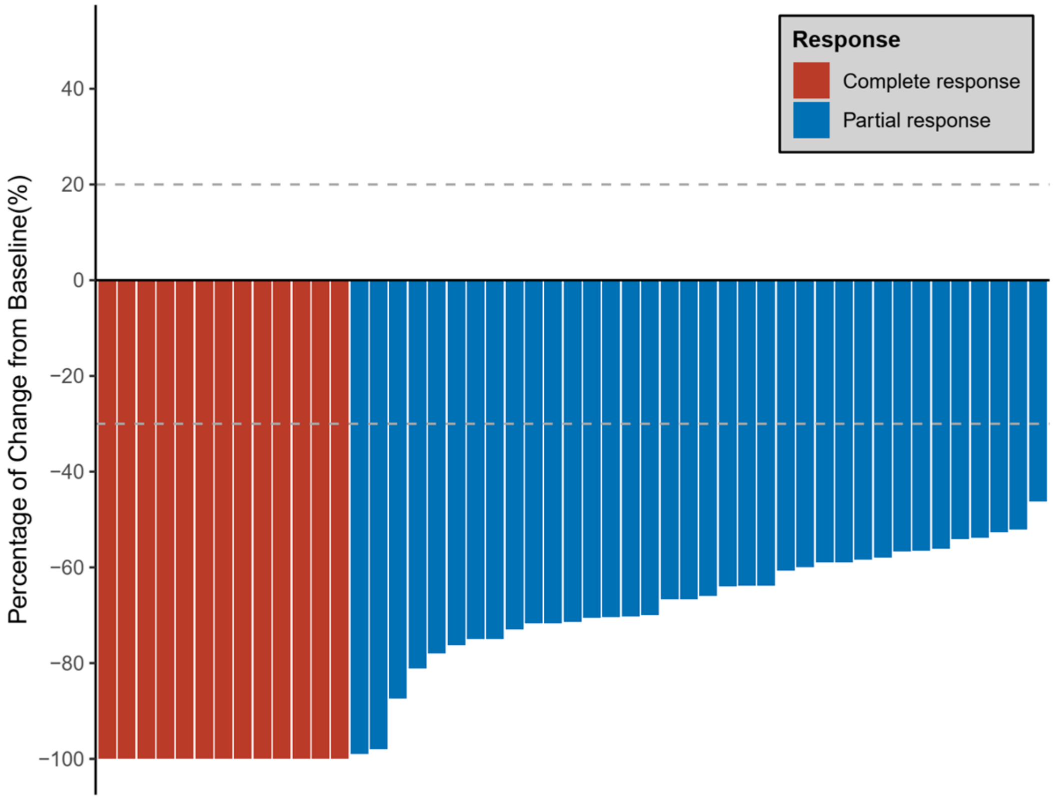

Supplementary Figure 2. Representative MRI comparison of pre- and after induction phase. Panel A (Right cavernous sinus invasion), B (Right oropharyngeal invasion), C (Right cavernous sinus, clivus, petrous apex, and area II-IV lymph node invasion) and D (Left cavernous sinus, retropharyngeal and area II-V lymph nodes invasion) denote nasopharyngeal and neck MRI before treatment. Panels E, F, G, and H denote complete response to induction therapy in the corresponding aforementioned sites, respectively.

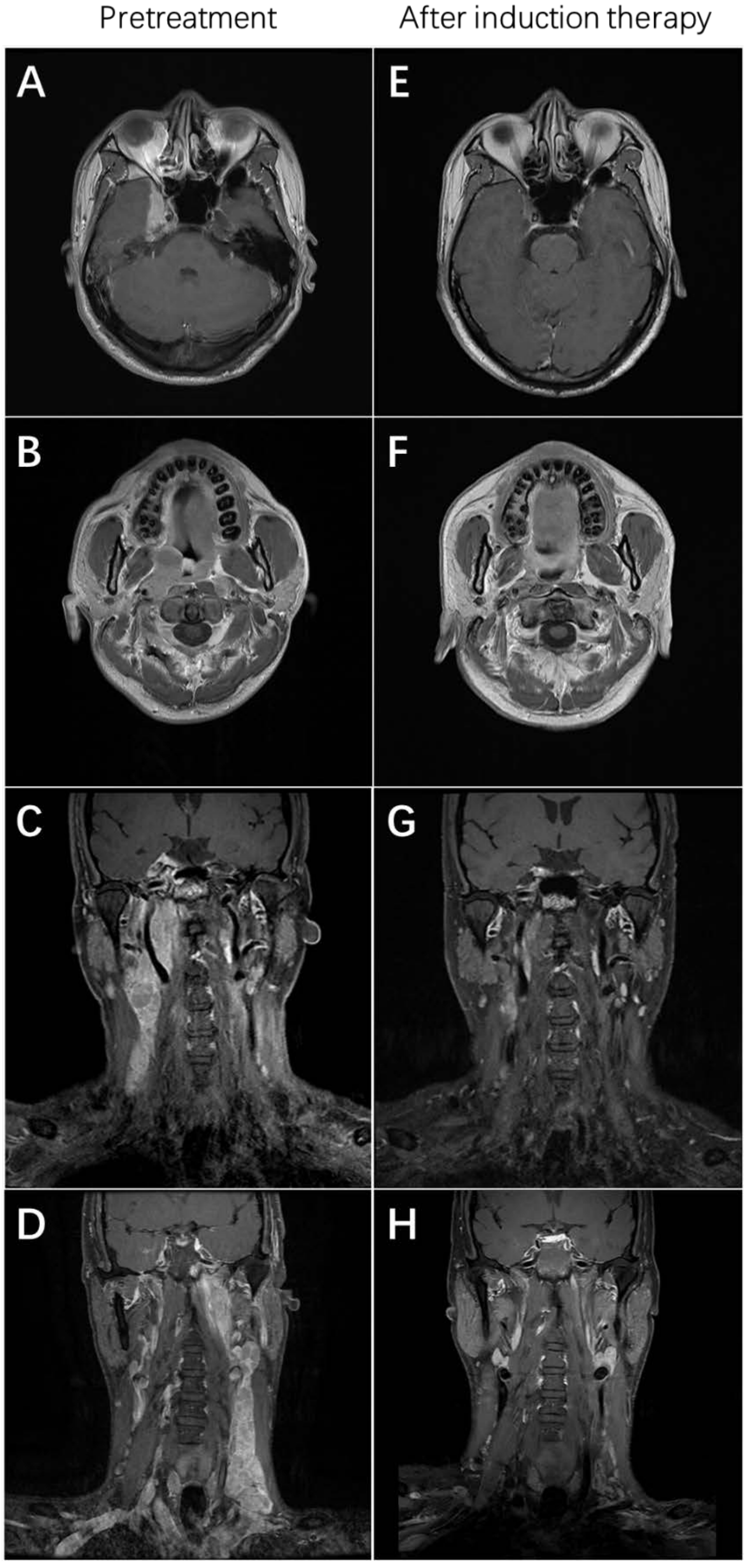

Supplementary Figure 3. Pathology of nasopharyngeal and lymph node tissues. Panels A and E denote hematoxylin-eosin staining of nasopharyngeal and lymph node tissue before treatment. Panels B and F denote immunohistochemistry staining of cell keratins (CK, positive) in nasopharyngeal and lymph node tissues before treatment, respectively. Panels C and G denote situ hybridization of Epstein-Barr encoding region (EBER, positive) in nasopharyngeal tissue and lymph node tissue before treatment, respectively. Panels D and H denote hematoxylin-eosin staining of nasopharyngeal and lymph node tissues after induction therapy showing pathological complete response, respectively.

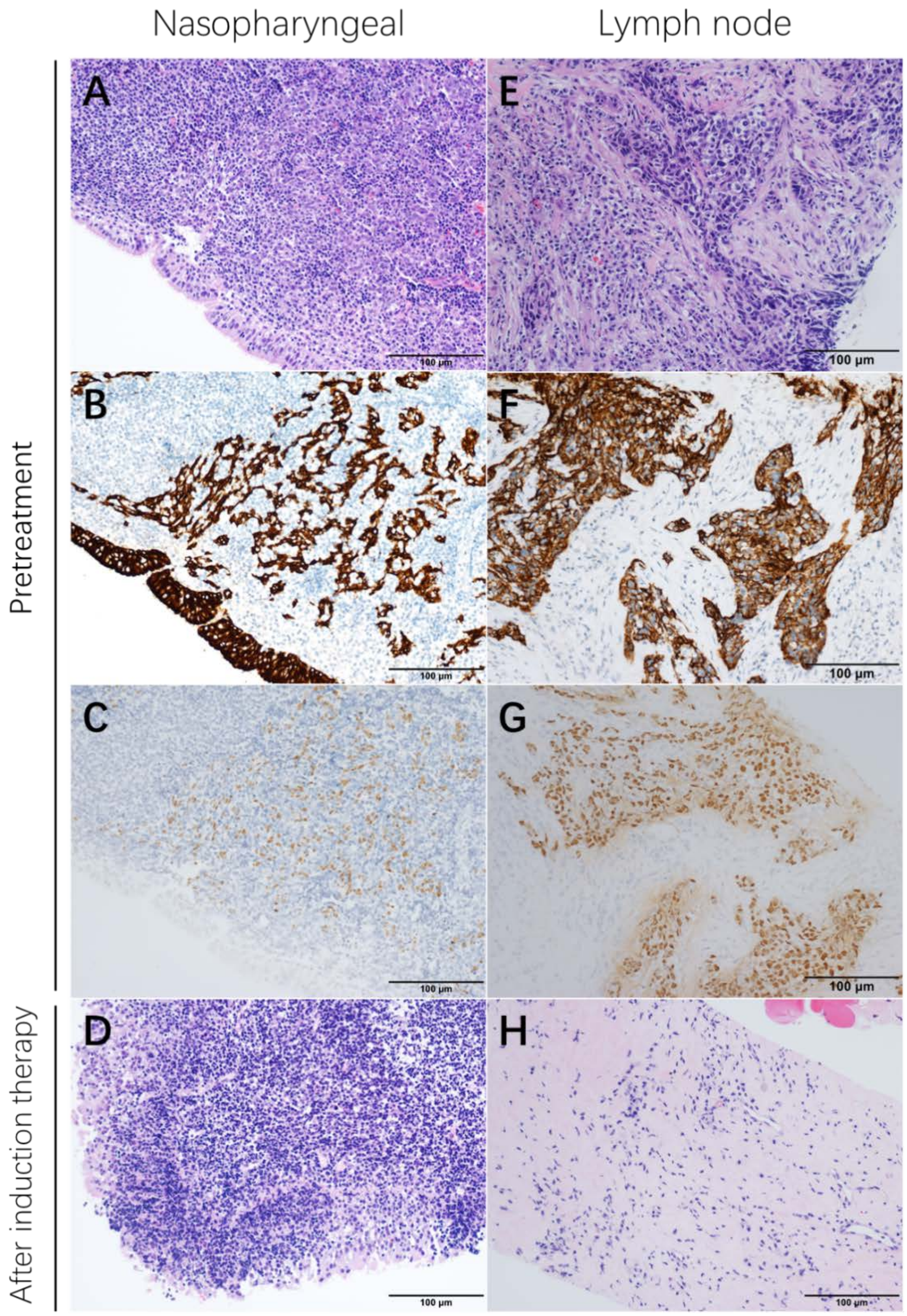

Supplementary Figure 4. Trajectories of plasma EBV DNA levels. The longitudinal monitoring trajectories of the change in plasma Epstein-Barr virus (EBV) DNA concentrations (n=48) in the intention-to-treat population. Red and orange dotted lines denote two patients that had persistently detectable plasma EBV DNA, including one achieving regional partial response and another dead from subsequent liver and bone metastases, respectively.

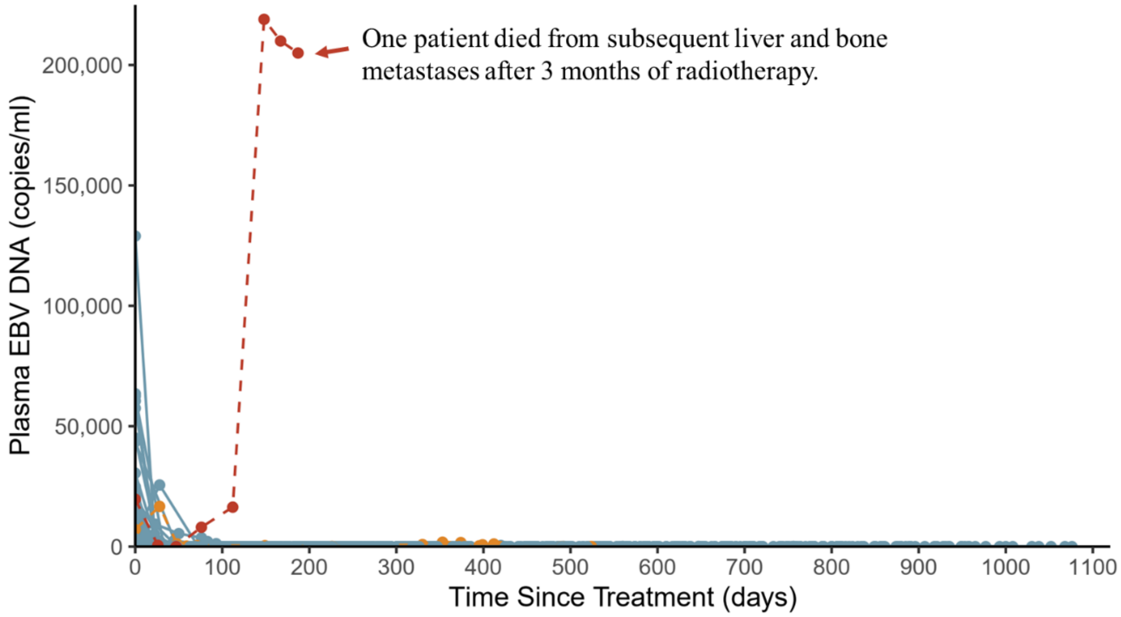

Supplementary Figure 5. Immune-related toxic epidermal necrolysis-like skin reaction. One 38-year-old male patient presented with rashes 2 weeks after receiving the first dose induction therapy (Panel A), and gradually developed toxic epidermal necrolysis (TEN)-like skin reaction with erythematous macules and pigmentation within 1 week (Panel B). This patient was hospitalized and received methylprednisolone 1 mg/kg/day and intravenous immune globulin for 7 days (Panel C). The skin rashes subsided after 10 days of hospitalization (Panel D). Panel E shows this patient 3 months after the last dose of camrelizumab. Panels F and G denote the histology of skin revealing intraepidermal edema and basal liquefaction with lymphocytic infiltration in epithelium and superficial dermis, respectively.*Authors have obtained consent from the patient to publish the images.

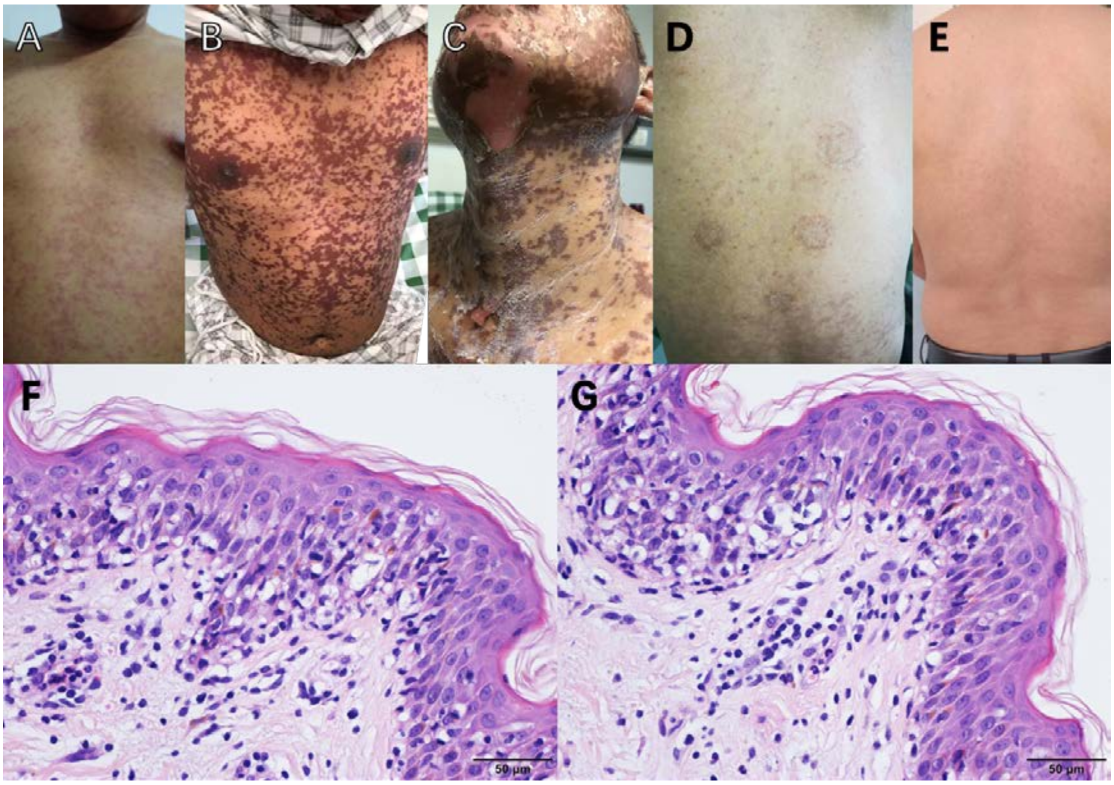

Supplementary Figure 6. Kaplan-Meier analysis of A distant metastasis-free survival; B failure-free survival; C overall survival; and D locoregional recurrence-free survival (intention-to-treat population). The primary end point was distant metastasis-free survival, defined as the time from the treatment initiation to the time of documented distant metastasis. Secondary end points included failure-free survival, overall survival, and locoregional recurrence-free survival. The rate of survival among the intention-to-treat population as compared with our historical cohort, which is the stage N3 subgroup of the study cohort described by Li et al (Li WZ, et al. JAMA Oncology 2022)[2]. Proportional-hazards assumption was tested with Schoenfeld residuals and did not hold. P values were evaluated by the two-sided log-rank test at the nominal 5% significance level. QUINTUPLED is the present trial code. Tick marks indicate censored data.

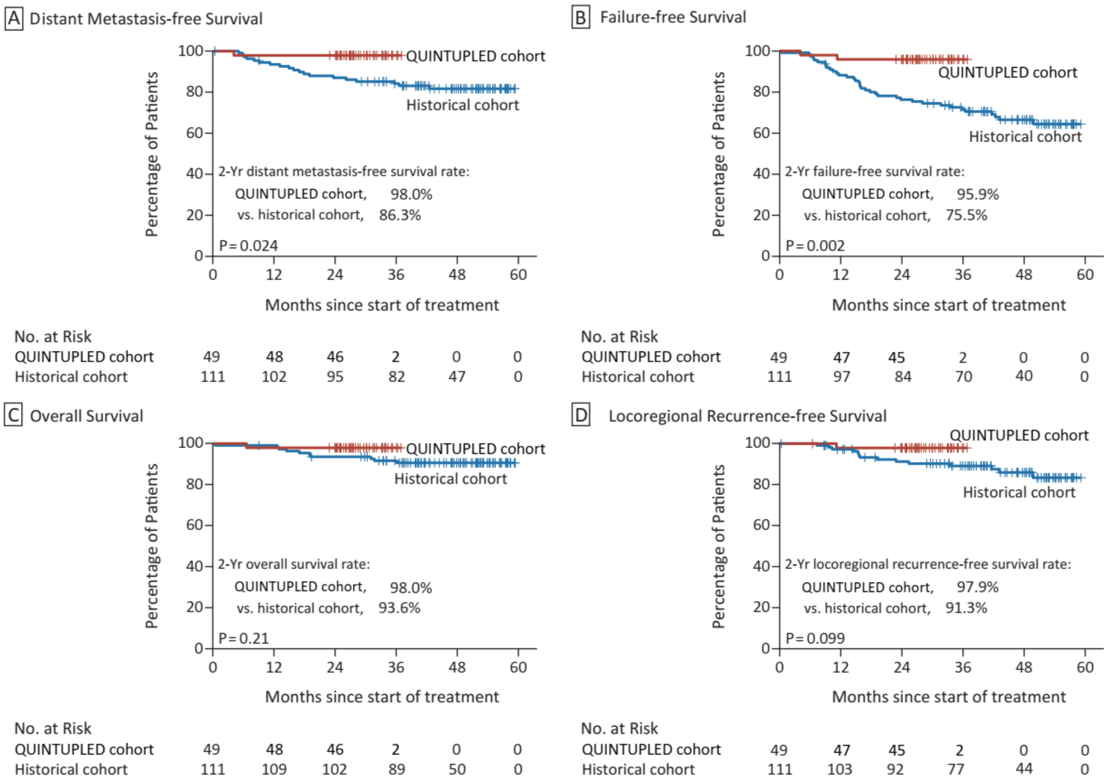

Supplementary References:

1. Eisenhauer EA, Therasse P, Bogaerts J, et al. New response evaluation criteria in solid tumours: revised RECIST guideline (version 1.1). Eur J Cancer 2009; 45: 228-47.
2. Li WZ, Lv X, Hu D, et al. Effect of induction chemotherapy with paclitaxel, cisplatin, and capecitabine vs cisplatin and fluorouracil on failure-free survival for patients with stage IVA to IVB nasopharyngeal carcinoma: a multicenter phase 3 randomized clinical trial. JAMA oncology 2022; 8:706-14.

Supplementary Note 1. Study Protocol

This supplement contains the following items:

1. Original protocol (*Page 32-70*), final protocol (*Page 71-108*), summary of changes (*Page 109*).

2. Original statistical analysis plan (*Page 110-112*), final statistical analysis plan (*same with the original version*), summary of changes (*no changes*).

Table of Contents

First approved version: April 25, 2020 ..... **32**

1 Background **33**

2 Objectives **34**

2.1 Primary Objectives 34

2.2 Secondary Objectives 34

3 Trial Scheme **35**

4 Subject Enrollment **36**

4.1 Eligibility Criteria 36

4.2 Exclusion Criteria 36

4.3 Criteria for withdrawal from protocol treatment 37

5 Treatment Plan **38**

5.1 Pretreatment Assessment 38

5.2 Pretreatment Staging Criteria 39

5.3 Chemotherapy, target and immunotherapy 39

5.4 Radiotherapy 41

5.4.1 Radiation Therapy 41

5.4.2 Target Volume Determination for IMRT 41

5.4.3 Prescribed Dose and Fractionation 42

5.4.4 Normal Tissue Dose Constraints 42

5.4.5 Radiotherapy Adjustments for Non-hematological Toxicity 43

5.4.6 Radiotherapy Adjustments for Hematological Toxicity 44

5.5 Salvage Therapy 44

6 Dosage Adjustment Principle for chemotherapy **44**

6.1 General principle for dosage adjustment 44

6.2 Dosage adjustment due to hematologic adverse events 45

6.3 Dosage adjustment due to non-hematologic adverse events 46

6.3.1 NAB-paclitaxel 46

6.3.2 Cisplatin 48

6.3.3 Capecitabin 48

6.3.4 Apatinib 49

6.3.5 Camrelizumab 51

7 Concomitant Medication or Treatment **53**

7.1 Application of Antiemetic Drugs 53

7.2 Application of Granulocyte Colony-stimulating Factor (G-CSF) 53

7.3 Combined drug usage and treatment 54

7.3.1 Permissible Treatment 54

7.3.2 Inadmissible Treatment 54

8 Observation and Assessment During Treatment **54**

9 Exploratory analysis and translational study **55**

9.1 Exploratory analysis 55

9.2 Translational study 55

10 Safety Evaluation **56**

10.1 Adverse Event Indicators 56
10.2 Severe Adverse Events (SAEs) 56
10.3 Record of Events 57
11 Follow Up 57
12 Safety Measures and Quality Control 58
13 Data Collection and Data Management 59
13.1 Case Report Form (CRF) 59
13.2 Data Entry and Modification 59
13.3 Data Locking 59
14 Statistical Analysis 60
14.1 Endpoint Definitions 60
14.1.1 Primary End Point 60
14.1.2 Secondary End Points 60
14.2 Determination of the Sample Size 62
14.3 Analytical Approach 62
15 Ethical Considerations 63
16 Administration of Experimental Drugs 63
17 Progress of Study 64
18 Principle center and investigator 64
19 Bibliography 65
Appendix I: The 8th editions of the AJCC Staging System of Nasopharyngeal Carcinoma 67
Appendix II: ECOG Performance Status and Scores 68
Appendix III: Acute Induction Chemotherapy and Chemoradiotherapy Toxicity Graded by Common Terminology Criteria for Adverse Events (CTCAE 5.0). 69
Appendix IV: Late Onset Toxicities Graded by RTOG/EORTC System 70
Final protocol (Second version: March 20, 2021) 71
1 Background 72
2 Objectives 73
2.1 Primary Objectives 73
2.2 Secondary Objectives 73
3 Trial Scheme 74
4 Subject Enrollment 75
4.1 Eligibility Criteria 75
4.2 Exclusion Criteria 75
4.3 Criteria for withdrawal from protocol treatment 76
5 Treatment Plan 77
5.1 Pretreatment Assessment 77
5.2 Pretreatment Staging Criteria 78
5.3 Chemotherapy, target and immunotherapy 78

5.4 Radiotherapy....80
5.4.1 Radiation Therapy....80
5.4.2 Target Volume Determination for IMRT....80
5.4.3 Prescribed Dose and Fractionation....81
5.4.4 Normal Tissue Dose Constraints....81
5.4.5 Radiotherapy Adjustments for Non-hematological Toxicity....82
5.4.6 Radiotherapy Adjustments for Hematological Toxicity....83
5.5 Salvage Therapy....83
6 Dosage Adjustment Principle for chemotherapy....83
6.1 General principle for dosage adjustment....83
6.2 Dosage adjustment due to hematologic adverse events....84
6.3 Dosage adjustment due to non-hematologic adverse events....85
6.3.1 NAB-paclitaxel....85
6.3.2 Cisplatin....87
6.3.3 Capecitabin....87
6.3.4 Apatinib....88
6.3.5 Camrelizumab....90
7 Concomitant Medication or Treatment....92
7.1 Application of Antiemetic Drugs....92
7.2 Application of Granulocyte Colony-stimulating Factor (G-CSF)....92
7.3 Combined drug usage and treatment....93
7.3.1 Permissible Treatment....93
7.3.2 Inadmissible Treatment....93
8 Observation and Assessment During Treatment....93
9 Exploratory analysis and translational study....94
9.1 Exploratory analysis....94
9.2 Translational study....95
10 Safety Evaluation....95
10.1 Adverse Event Indicators....95
10.2 Severe Adverse Events (SAEs)....96
10.3 Record of Events....96
11 Follow Up....97
12 Safety Measures and Quality Control....98
13 Data Collection and Data Management....98
13.1 Case Report Form (CRF)....98
13.2 Data Entry and Modification....98
13.3 Data Locking....99
14 Statistical Analysis....99
14.1 Endpoint Definitions....99
14.1.3 Primary End Point....99
14.1.4 Secondary End Points....99
14.2 Determination of the Sample Size....101
14.3 Analytical Approach....101

15 Ethical Considerations 102
16 Administration of Experimental Drugs 102
17 Progress of Study 103
18 Principle center and investigator 103
19 Bibliography 104
Appendix I: The 8th editions of the AJCC Staging System of Nasopharyngeal Carcinoma 105
Appendix II: ECOG Performance Status and Scores 106
Appendix III: Acute Induction Chemotherapy and Chemoradiotherapy Toxicity Graded by Common Terminology Criteria for Adverse Events (CTCAE 5.0). 107
Appendix IV: Late Onset Toxicities Graded by RTOG/EORTC System 108
Summary of changes in protocol 109
Original statistical analysis plan 110

Efficacy and Safety of Camrelizumab and Apatinib Combined with Induction Chemotherapy and Concurrent Chemoradiotherapy for Stage TanyN3M0 Nasopharyngeal Carcinoma: A Phase II QUINTUPLED Trial

First approved version: April 25, 2020
Trial Code: QUINTUPLED

**Principal Investigator**	Dr. Yan-Qun Xiang<br>Department of Nasopharyngeal Carcinoma<br>Cancer Center, Sun Yat-Sen University<br>651 Dongfeng Road East, Guangzhou, China<br>Tel: (020)-87343379; Fax: (020)-87343379<br>E-mail: xiangyq@mail.sysu.edu.cn
**Participating Center**	
Sun Yat-Sen University Cancer Center, Guangzhou, China	
Lead investigators	Dr. Yan-Qun Xiang<br>Dr. Chong Zhao<br>Department of Nasopharyngeal Carcinoma<br>Dr. Changqing Xie<br>Thoracic and GI Malignancies Branch<br>National Cancer Institute, NIH
**Data Manager & Statistician**	Dr. Qing Liu<br>Department of Medical Statistics and Epidemiology, Cancer Center, Sun Yat-Sen University<br>Dr. Yan-Fang Ye<br>Clinical Research Design Division, Sun Yat-Sen Memorial Hospital, Sun Yat-Sen University
**Honorary Adviser**	Dr. Xiang Guo<br>Dr. Hai-Qiang Mai<br>Department of Nasopharyngeal Carcinoma<br>Cancer Center, Sun Yat-Sen University

Activation Date

First approved version: April 25, 2020

Current Edition: April 25, 2021

1 Background

Nasopharyngeal carcinoma (NPC) differs from other head and neck cancers with unique geographical, etiological, and biological features.[1] It is endemic in the area of Southern China, Southeast Asia, and North Africa. More than 70% of NPC patients are classified as having locoregionally advanced disease among the 129,000 newly diagnosed cases annually.[1] Induction chemotherapy followed by concurrent chemoradiotherapy is the current standard treatment for locoregionally advanced NPC, recommended by the National Comprehensive Cancer Network (NCCN) Guidelines as level 1 evidence. With the application of MRI, intensity-modulated radiotherapy and combined chemoradiotherapy has substantially improved locoregional control. However, distant metastasis predominates as the pattern of treatment failure. In particular, approximately 50% of patients with T_{any}N3M0 diseases eventually develop distant metastasis after undergoing abovementioned radical treatment. Therefore, more effective treatment strategies are needed for this patient population.

Due to the properties of NPC with the abundant lymphocytic infiltrations and elevated expressions of programmed death-ligand 1 (PD-L1)[2], immune checkpoint blockade therapy has considerable therapeutic potential for patients with NPC. Its effect on the treatment has confirmed in recurrent or metastatic NPC (RM-NPC) in the first-, second-, and greater-line settings.[3-6] However, there are no available reports assessing the value of adding immunotherapy to standard treatment in the curative setting to date.

Camrelizumab (SHR-1210) is a fully humanized immunoglobulin G4/k PD-1 monoclonal antibody, which is well tolerated with positive antitumor activity in RM-NPC. At the same time, antiangiogenic therapies have been shown to sensitize anti-PD-1/PD-L1 treatment by increasing PD-L1 expression and CD8+ T cell infiltration in preclinical models.[7,8] Remarkably, apatinib, an orally administered vascular endothelial growth factor receptor 2 (VEGFR2) tyrosine kinase inhibitor, has demonstrated antitumoral activity and synergetic effects with camrelizumab in several solid tumors.[9-12] Therefore, we evaluated the effect and safety of the combination of camrelizumab

and apatinib with standard treatment regimen as the curative setting in stage $T_{any}N3M0$ NPC.

2 Objectives

2.1 Primary Objectives

To assess distant metastasis-free survival of camrelizumab plus apatinib combined with induction chemotherapy (TPC; NAB-paclitaxel, cisplatin and capecitabine) and concurrent chemoradiotherapy for high-risk of metastatic NPC patients (stage $T_{any}N3M0$).

2.2 Secondary Objectives

To evaluate response rate, overall survival (OS), failure-free survival (FFS) rate, locoregional recurrence-free survival (LRFS), toxicity profile, treatment adherence of camrelizumab plus apatinib combined with induction chemotherapy (TPC; NAB-paclitaxel, cisplatin and capecitabine) and concurrent chemoradiotherapy for high-risk of metastatic NPC patients (stage $T_{any}N3M0$).

3 Trial Scheme

Nasopharyngeal carcinoma
Histology: non-keratinizing
Staging: TanyN3M0 by AJCC 8th system*

↓

Induction phase
3 cycles of Camrelizumab and Apatinib combined with induction chemotherapy (TPC; NAB-paclitaxel, cisplatin and capecitabine)

↓

Concurrent chemoradiotherapy phase
Intensity-modulated radiotherapy with 2 cycles of concurrent cisplatin and Camrelizumab every 3 weeks

↓

Maintenance phase
Camrelizumab every 3 weeks up to 1 year

↓

Primary endpoint: Distant metastasis-free survival
Secondary endpoint: Treatment response, toxicity profile, overall survival, failure-free survival, local relapse-free survival, treatment adherence

*The 8th edition of the American Joint Commission on Cancer staging system.

4 Subject Enrollment

4.1 Eligibility Criteria

a) Patients newly diagnosed with histologically confirmed differentiated or undifferentiated non-keratinized carcinomas (according to the world health organization [WHO] histologically type).

b) Patients with tumors staged as $T_{any}N3M0$ (as defined by the 8th AJCC edition, appendix I).

c) No sign of distant metastasis.

d) Without previous anticancer treatment.

e) Without a history of other malignancies.

f) Age between 18-65 years.

g) Adequate bone marrow function: White blood cell count of $\geq 4.0\times10^9$/L, absolute neutrophil count (ANC) of $\geq 2.0\times10^9$/L, platelet count of $\geq 100\times10^9$/L, and hemoglobin concentrations of ≥ 90 g/L.

h) Sufficient renal function: creatinine clearance rate at greater than or equal to 60 mL/min.

i) Normal liver function: aspartate aminotransferase (AST) and alanine aminotransferase (ALT) ≤ 2.0 times the upper limit of normal (ULN).

j) Eastern Cooperative Oncology Group performance status of 0 or 1.

k) Signed informed consent form.

4.2 Exclusion Criteria

a) Patients suffering from basaloid squamous cell carcinoma or WHO Type keratinizing squamous cell carcinoma.

b) Previous anticancer treatment, including chemotherapy, radiotherapy, and surgery (except diagnostic).

c) The presence of distant metastasis at initial diagnosis.

d) Patients who are pregnant or lactating (in women of child-bearing age, pregnancy test should be considered and effective contraception during the treatment period should be emphasized).

e) Patients suffering from any severe uncontrollable infections, serious physical or mental diseases or abnormal laboratory tests, which may incur unacceptable risk, negatively affect trial compliance, or affect the administration, distribution, metabolism, and excretion of study drugs. For example, unstable cardiac disease necessitating treatment, chronic hepatitis renal disease, poorly controlled diabetes (fasting plasma glucose greater 1.5 × the ULN), emotional disturbance, mental disorders, abnormal central nervous system, chronic diarrhea, ascites, and pleural effusion.

f) Hypertension, and cannot be well controlled by antihypertensive drugs (systolic blood pressure > 140 mmHg or diastolic blood pressure > 90 mmHg).

g) Human immunodeficiency virus (HIV) infection or known acquired immunodeficiency syndrome (AIDS), untreated active hepatitis (hepatitis B is defined as HBV-DNA ≥ 500 IU/ml; hepatitis C is defined as HCV-RNA higher than the detection limit of the analytical method) or co-infection of hepatitis B and C.

h) Patients who cannot cooperate with routine follow-up because of psychological, social, family, or geographical reasons.

i) Patients with a history of other malignancies before admission.

j) Patients with a history of psychotropic substance abuse or drug use.

k) Receive experimental treatment from other clinical studies at the same time (during the treatment period of this trial).

l) Refuse to sign the informed consent form.

m) Long-term use of immunosuppressive agents after organ transplantation.

4.3 Criteria for withdrawal from protocol treatment

The reasons for subjects' withdraw from the study may include:

a) Evidence of disease recurrence.
b) The patient may withdraw from the study and reject further follow-up at any time for any reason. The reason should be recorded.
c) Unacceptable toxicity, the reasons for which must be recorded.
d) The test results of the subject's β-HCG test indicated that the subject was pregnant. At the same time, pregnancy will also be reported as a serious adverse event reporting procedure.
e) Use of prohibited medications or other medications that the investigator believes will cause a toxic reaction or affect the results of the study.
f) Patients suffering from an intercurrent disease that significantly affects the assessment of clinical status or necessitates discontinuation of the drug, or both.
g) Investigators believe that the termination of treatment is beneficial to patients according to their physical condition.
h) Death.

5 Treatment Plan

5.1 Pretreatment Assessment

All patients are under standardized management for NPC, and they need to perform a series of examinations as well as provide relevant information to confirm pathologic diagnosis and clinical stage before admitted into trial:

a) Signed informed consent.
b) Medical history review.
c) Personal data collection.
d) Current medications and treatment.
e) Body examinations, include height, weight and vital signs.
f) Physical examination of head and neck region, include nasopharynx and cervical lymph nodes.
g) Physical examination of the nervous system.

h) Nasal endoscopy and lesion biopsy.
i) Complete blood count and differential, electrolytes and liver function test.
j) Urinalysis.
k) EBV antibodies and EBV DNA testing.
l) Virological examination (completed within 14 days prior to the first dose): HBsAg (if positive, HBV-DNA is required), HBsAb, HBeAg, HBeAb, HBcAb, HCV-Ab (if positive, HCV-RNA is required) , HIV-Ab.
m) Thyroid function.
n) Coagulation function.
o) Electrocardiogram and Echocardiography.
p) Imaging, including enhanced MRI or enhanced CT of the head and neck (CT is indicated only in patients with contraindication to MRI).
q) Chest CT.
r) ECT bone scan.
s) Abdominal CT/MR.
t) PET/CT is optional and is performed at the discretion of the attending physician.
u) Pregnancy test (applicable to women of childbearing age).

5.2 Pretreatment Staging Criteria

The disease stage of subjects will be staged according to the 8th edition of the Union for International Cancer Control / American Joint Committee on Cancer (UICC/AJCC) staging system (Appendix I).

5.3 Chemotherapy, target and immunotherapy

5.3.1 Induction therapy phase

1. Camrelizumab

Camrelizumab was intravenously given at dose of 200 mg, administered as a 30-minutes (no less than 20 minutes, no more than 60 minutes) infusion on day 1.

Camrelizumab will be administrated throughout the trial every three weeks up to one year.

2. Induction chemotherapy with TPC regimen

The TPC regimen consisted of NAB-paclitaxel at a dose of 200 mg per square meter, administered over 30 minutes infusion on day 1, followed by cisplatin at a dose of 60 mg per square meter on day 1; No premedication against allergic reaction was given before the NAB-paclitaxel; And capecitabine at a dose of 1000 mg per square meter, taken orally twice a day on days 1 to 14; repeat every 21 days for 3 cycles.

After the infusion of camrelizumab, NAB-paclitaxel and cisplatin are successively administered intravenously after an interval of at least 30 min.

3. Apatinib

Apatinib taken orally at a dose of 250 mg daily for a total of 8 weeks. The drug should be administered on the first day of initial treatment and discontinued one week before radiotherapy.

5.3.2 Concurrent chemotherapy with cisplatin

Concurrent chemotherapy with cisplatin

The concurrent chemotherapy consisted of cisplatin at a dose of 100 mg per square meter, administered as a 4-hour infusion; repeat every 21 days for 2 cycles.

Cisplatin administration follows local hydration protocols to prevent/minimize nephrotoxicity. Pre-hydration (at least 12 hours before cisplatin administration, D0) and post-hydration (D1-3) with over 2000 ml of normal saline infused each time are both needed. The use of mannitol (D1) and furosemide (D1) is essential for it helps to reduce cisplatin-related nephrotoxicity by diuresis. Twenty-four hours urinary output should be recorded (D0-D3) and urinalysis will be performed (D2-D3). Electrolytes and serum creatinine are monitored during treatment and a follow-up creatinine within 24-48 hours of the cisplatin dose is recommended. One hour before concurrent chemotherapy, patients are given 5-hydroxytryptamine-3 receptor antagonists for anti-nausea (D1). The NK-1 receptor antagonist aprepitant QD were recommended in D1-D3.

5.4 Radiotherapy

5.4.1 Radiation Therapy

Radiation therapy with intensity-modulated radiotherapy (IMRT) techniques is mandatory. Target volumes are defined following the International Commission on Radiation Units and Measurements (ICRU) reports 50 and 62. The principle of target volume determination for IMRT and prescribed dose and fractionation areas are described as follows.

5.4.2 Target Volume Determination for IMRT

Gross tumor volume (GTV)

(1) Gross tumor target volume of primary nasopharyngeal focus (GTVnx): primary nasopharyngeal tumors and their invasive areas revealed by clinical examination and imaging examination.

(2) Clinically involved gross cervical lymph nodes (GTVnd): cervical metastatic lymph nodes clinically affected and/or imaged (imaging diagnostic criteria: a. short diameter >1 cm; b. necrotic focus in the center; c. extracapsular invasion; d. clustered or clustered short diameter >0.8 cm).

Clinical target volume (CTV)

(1) Nasopharyngeal clinical target volume I (CTV1, nasopharyngeal high-risk area): GTVnx expands 0.5-1.0 cm forward, upper, lower, and bilateral, and 0.2-0.3 cm backward (according to the characteristics of adjacent tissue structure), which should also include all nasopharyngeal mucosa and 0.5 cm below.

(2) Clinical target volume II (CTV2, nasopharyngeal low-risk area, cervical metastatic lymph node area, and cervical area requiring preventive irradiation): CTV1 expands 0.5-1.0 cm forward, upper, lower, and bilateral, 0.2-0.3 cm backward (according to adjacent tissue structure characteristics) including the lymphatic drainage area of GTVnd and extends to negative lymph node drainage

area requiring preventive irradiation (Table 1).

**Table 1.** Cervical preventive exposure areas	
Lymph node metastasis	Cervical lymph node area (CTV2 neck) requiring radiation prevention
II	Ipsilateral II, III, IV, Va, Vb
III	Ipsilateral II, III, IV, Va, Vb
Va	Ipsilateral II, III, IV, Va, Vb
IV	Ipsilateral II, III, IV, Va, Vb, supraclavicular region
Vb	Ipsilateral II, III, IV, Va, Vb, supraclavicular region
Unilateral neck	Contralateral zones II, III, Va

Note: Indications of area are included in CTV2: (1) metastatic lymph nodes in area IIa (> 3 cm); (2) extracapsular invasion of metastatic lymph nodes in area IIa; (3) the metastatic lymph nodes in area IIa push the submandibular gland or have no clear demarcation with the submandibular gland; (4) lymph node metastasis in multiple areas of the ipsilateral whole neck (>4 areas).

Planned target volume (PTV)

PTVnx, PTVnd, PTV1, and PTV2 are GTVnx, GTVnd, CTV1, and CTV2, respectively, with expansion to a certain distance, generally 0.3 cm in the forward, upper, lower, left and right directions, and 0.1-0.2 cm in the backward direction.

5.4.3 Prescribed Dose and Fractionation

All patients will be treated with IMRT using simultaneously integrated boost, 5 fractions per week. The prescribed dose is PTVnx: 68-72 Gy/30-33F, PTVnd: 64-68 Gy/30-33F, PTV1: 60-64 Gy/30-33F, PTV2: 54-58 Gy/30-33F, for the PTVs derived from GTVnx, GTVnd, CTV1, and CTV2, respectively.

5.4.4 Normal Tissue Dose Constraints

**Table 2.** Normal tissue dose constraint structure.	
**Structure**	**Dose constraints**

**Critical organ at risk**	
Brain stem	Dmax ≤ 54 Gy
PRV-Brain stem	D1 ≤ 60 Gy
Spinal cord Dmax*	Dmax* ≤ 45 Gy
PRV-Spinal cord	D1†≤ 50 Gy
Optic nerve	Dmax ≤ 54 Gy
PRV-Optic nerve	D1 ≤ 60 Gy
Optic chiasm	Dmax ≤ 54 Gy
PRV-Optic chiasm	D1 ≤ 60 Gy
Temporal lobe	Dmax ≤ 60 Gy
PRV-Temporal lobe	D1 ≤ 65 Gy
**Intermediate-risk organ at risk**	
Pituitary	Dmax < 60 Gy
Mandibular	Dmax < 70 Gy
Temporomandibular Joint	Dmax < 70 Gy
Lens	Dmean‡ < 8 Gy
Eyes	Dmean < 35 Gy
**Low-risk organ at risk**	
Parotid	Dmean < 26 Gy
Parotid	V30¶ < 50%
Cochlea	Dmean < 50 Gy
Tongue	Dmean < 45 Gy
Larynx	Dmean < 45 Gy

PRV = planning organ at risk volume.
* Maximum point dose to the target volume.
† Dose received by 1% of the target volume.
‡ Mean dose to the target volume.
¶ At least 50% of the gland will receive <30 Gy (should be achieved in at least one gland)

5.4.5 Radiotherapy Adjustments for Non-hematological Toxicity

Side effects of radiotherapy may include anorexia, mucositis and skin reaction. The investigator will manage these conditions according to clinical practice. No radiotherapy dose modifications are allowed. Treatment interruptions are allowed if symptomatic mucositis or skin reactions occur that, in the judgment of the attending clinician, warrants a break. The treatment is completed according to protocol for treatment breaks up to and including 14 days. If the break exceeded 14 days, the patient

is removed from protocol treatment, completing treatment at the discretion of his or her physician but followed up and included in the analysis.

5.4.6 Radiotherapy Adjustments for Hematological Toxicity

Radiotherapy will be withheld until ANC > 0.5×10^9/L and platelet > 25×10^9/L.

5.5 Salvage Therapy

Nasopharyngeal surgery, neck dissections, secondary radiotherapy, chemotherapy, or immunotherapy may be given to patients with relapse or metastasis after treatment according to guidelines of National Comprehensive Cancer Network (NCCN).

6 Dosage Adjustment Principle for chemotherapy

6.1 General principle for dosage adjustment

(1) Assessment and grading for toxicity in patients will be carried out using the the Common Terminology Criteria for Adverse Events, version 5.0, of the National Cancer Institute (NCI CTCAE V5.0).

(2) There will be no dose escalation of NAB-paclitaxel, cisplatin and capecitabine (TPC) regimen.

(3) Chemotherapy dosage modifications based on nadir counts and interim nonhematological toxicities from the preceding cycle.

(4) Dosage may not be reduced or discontinued if the investigator considers that the adverse reaction is unlikely to develop into a serious or life-threatening events and will not result in delay or interruption of treatment. There is no need to reduce the dosage or discontinue treatment if anemia can be well alleviated by blood transfusion.

(5) A delay of up to 3 weeks for a cycle will be permitted to allow for the severity of toxic events of grade 3/4 to be reduced to grade 1 or less (except for alopecia, fatigue, malaise, and nail changes). Delays in excess of 3 weeks will necessitate require discontinuation of chemotherapy unless continued therapy is deemed necessary by the investigator.

(6) Chemotherapy must be withheld until the neutrophil count is > 1.5×10^9/L and the platelet count is > 100×10^9/L.

(7) Missing capecitabine doses due to toxicity should be no longer replenished or resumed, and the patient continued the scheduled course of treatment instead.

(8) If creatinine clearance was less than 70 ml/min but greater than 50 ml/min at any time during the previous chemotherapy course. The dose of cisplatin should be reduced by 50% during the next cycle of chemotherapy. If the creatinine clearance rate was less than 50 ml/min during the previous chemotherapy course, subjects should discontinue study therapy.

6.2 Dosage adjustment due to hematologic adverse events

The hematological toxicity is assessed according to the NCI CTC AE V5.0 classification standard, and the dosage adjustment of NAB-paclitaxel and cisplatin is performed according to the following principles.

Dosage adjustment of NAB-paclitaxel and cisplatin for hematologic toxicity[a]				
Toxicity	Grade	Time	NAB-paclitaxel	Cisplatin
Anemia	Any	Any	Full dosage	Full dosage
Neutropenia[b]	Grade 3	Any	Full dosage	Full dosage
	Grade 4	First time	160 mg/m^2	50 mg/m^2
		Second time	120 mg/m^2	40 mg/m^2
		Third time	Stop	Stop
Neutropenic fever[c]	Grade 3-4	First time	160 mg/m^2	50 mg/m^2

		Second time	120 mg/m^2	40 mg/m^2
		Third time	Stop	Stop
Thrombocytopenia	Grade 4	First time	160 mg/m^2	50 mg/m^2
		Second time	120 mg/m^2	40 mg/m^2
		Third time	Stop	Stop

Note:

a. Grade 3 neutropenia or thrombocytopenia, which does not result in prolonged chemotherapy interval, does not require dosage reduction. If this leads to prolonged chemotherapy interval, G-CSF or platelet-enhancing drug support may be considered for the next course of treatment. If this is still not resolved, the dosage should be reduced with the reference to the corresponding grade 4.

b. Grade 3 or 4 neutropenia may be consideration for prevention with G-CSF support during the next course of treatment.

c. Grade 3 or 4 neutropenic fever may be prevented with G-CSF support while the dosage is reduced during the next cycle of treatment. The definition of neutropenic fever: ANC < 1.0×10^9/L with fever ≥ 38.5 ℃ for grade 3 and ANC < 1.0×10^9/L with fever ≥ 38.5 ℃ with life-threatening sepsis for grade 4.

6.3 Dosage adjustment due to non-hematologic adverse events

6.3.1 NAB-paclitaxel

Peripheral neuritis and extremities joint myalgia

If a patient develops one of the above symptoms, the following methods are recommended to adjust the dosage of NAB-paclitaxel according to the duration and severity of the symptoms. Symptomatic treatment with non-steroidal anti-inflammatory drugs can be given alleviate extremities joint myalgia.

Dose adjustment of NAB-paclitaxel for non-hematologic toxicity

Adverse events	Grade	Duration of adverse events		
		1-7 days	Over 7 days	Persistent during treatment
Pain/paresthesia/sensory disturbances, but does not affect function	1	Full dose	Full dose	160 mg/m^2
Pain/paresthesia/sensory disturbances that affect function but do not affect daily activities	2	Full dose	Full dose	120 mg/m^2
Pain/paresthesia/sensory disturbances, with functional impairment, and interference with daily activities	3	Full dose	120 mg/m^2	Stop
Pain/paresthesia/sensory disturbances resulting in loss of function or life threating	4	Stop	Stop	Stop

Abnormal bilirubin and impaired liver function

For HBs-Ag positive or serum HBV DNA positive patients, prophylactic anti-HBV drugs should be used before treatment. If the liver function damage after chemotherapy is caused by the activation of hepatitis B virus, anti-hepatitis B virus drug therapy should be strengthened, and positive symptomatic treatment should be carried out. If grade 2 liver toxicity occurs, there is no need for adjusting NAB-paclitaxel dosage, but if grade 3 liver toxicity occurs, NAB-paclitaxel dosage should be reduced by 20%. Whenever grade 4 liver toxicity occurs, NAB-paclitaxel should be permanently discontinued. If grade 2-4 toxicity occurs, medication will be suspended until the toxicity returns to grade 1 or below before restarting treatment.

Other toxicity

If other grade 3 or less toxic reactions occur, symptomatic treatment should be given as far as possible, with no reduction of NAB-paclitaxel for the next course of treatment. If grade 3 toxicity occurs, when the toxicity reverts from grade 3 to grad 1 or below, the dosage of NAB-paclitaxel for the next course of treatment is reduced by 20%. If grade 4 toxicity occurs, treatment with NAB-paclitaxel should be permanently

discontinued. However, in the event of certain grade 3 or higher adverse events (i.e., fatigue, changes in sexual function, and dry skin without clinical significance), the investigator may continue the treatment at her or his discretion.

6.3.2 Cisplatin

Other non-hematological toxicity

If other toxicity reaction less than grade 3 occur, symptomatic treatment should be given whenever possible. If grade 3 or 4 toxicity occurs, treatment should be suspended, and when toxicity reverts to grade 1 or below, cisplatin should be administered at 80% of the initial dosage for the next course of treatment.

6.3.3 Capecitabin

Once the dosage of capecitabin has been reduced, it should not be increased later. Dosage adjustment is not recommended when grade 1 adverse events occur. Capecitabin treatment should be suspended if grade 2 or 3 adverse events occur. Treatment can be restarted with the original dosage of capecitabin or at the dosage adjusted in the following principles once the adverse reaction has disappeared or the severity has reduced to grade 1. If grade 4 adverse reactions occur, treatment should be suspended until the adverse reactions disappear or the severity is reduced to grade 1, and then treatment should be restarted at 50% of the original dosage. Missing capecitabine doses due to toxicity should be no longer replenished or resumed, and the patient continued the scheduled course of treatment instead.

The detailed dose adjustment of capecitabine is performed according to the following principles.

Table 3: Dose adjustment of capecitabine.

NCI assessed advent events	During treatment	Dose of the next cycle (% original dose)
**Grade 1**	Full dose	Full dose
**Grade 2**		

First time	Suspended until returned to grade 0~1	100%
Second time	Suspended until returned to grade 0~1	75%
Third time	Suspended until returned to grade 0~1	50%
Fourth time	Permanent termination	
**Grade 3**		
First time	Suspended until returned to grade 0~1	75%
Second time	Suspended until returned to grade 0~1	50%
Third time	Permanent termination	
**Grade 4**		
First time	Permanent termination or suspended until returned to grade 0~1 if researcher believe it is in the patient's interest to continue treatment	50%
Note: patients with hand-foot syndrome would be recommended treated with topical emollient, topical corticosteroid cream, or creams containing urea.		

6.3.4 Apatinib

Adverse reactions should be closely monitored during treatment and the dose should be adjusted as needed to enable patients to tolerate the treatment. Once the dose of the drug has been reduced, it should not be increased later. Apatinib 250 mg is recommend orally administrated daily, 7 days every week. In case of adverse reactions related to apatinib, dose frequency should be adjusted according to the following conditions: The recommended frequency should be reduced to:

- 250 mg daily, 5 days every week for the first adjustment;
- 250 mg every two days for the second adjustment;
- Suspended if the relative adverse events occurred third time; After the drug reaction disappeared, the dose before the adjustment could be restored.
- If the patient is still intolerant, stop apatinib permanently.

Hypertension

For patients with hypertension, antihypertensive drugs should be used reasonably before the initiation of apatinib administrated, and blood pressure should be closely monitored during treatment. For patients with grade 1 or 2 hypertension (120-159 mmHg systolic pressure or 80-99 mmHg diastolic pressure) during treatment, there is no need to adjust the dose of apatinib; For grade 3 hypertension (systolic pressure ≥ 160mmHg or diastolic pressure ≥ 100mmHg), apatinib should be stopped. After drug management intervention, apatinib can be continued with reduced dosage if blood pressure is well controlled. Patients with grade 4 hypertension, such as hypertensive crisis and other malignant hypertensive reactions, need to stop taking apatinib immediately and permanently.

Proteinuria

Urine protein should be monitored closely during treatment. Urine routines and/or 24-hour proteinuria are recommended every 2 weeks for the first 2 months and every 4 weeks thereafter. There is no need to adjust the dose of apatinib for grade 1 or 2 urine protein during the treatment. Apatinib should be stopped for grade 3 (24-hour urine protein quantification ≥ 3.5 g), and drug intervention should be performed. After proteinuria is restored to grade 2 or below, apatinib can be continued with reduced dosage. If grade 3 proteinuria occurs again after twice reduction, apatinib should be stopped permanently.

Hand and foot skin reaction

It is recommended that the hands and feet skin reaction drug prevention intervention can be applied with urea ointment, moisturizing cream or aloe gel on the skin of hands and feet from the first day of taking apatinib. For grade 1 reaction, local symptomatic support is recommended without dose adjustment; For grade 2 reaction, dose can be adjusted appropriately; For grade 3 reaction, apatinib should be suspended; After symptomatic support, apatinib can be continued with reduced dosage; if the reaction persist or aggravate, apatinib should be discontinued.

Bleeding or thrombosis

For grade 2 venous thrombosis, symptomatic treatment and close monitoring was

recommended without dose adjustment; For grade 3 or asymptomatic grade 4 reaction, apatinib should be stopped and treated with anticoagulation for at least one week. When the symptoms of thrombosis improve and grade 3-4 bleeding does not occur, apatinib can be continued with adjusted dose, otherwise the drug should be stopped.

Gastrointestinal reaction

For patients with grade 1 reaction, local symptomatic support is recommended without dose adjustment. For grade 2 reaction, especially diarrhea, vomiting and other symptoms, apatinib should be suspended if symptoms did not relieve after symptomatic treatment; For grade 3-4 reaction, apatinib should be discontinued immediately.

Impaired liver function

For HBs-Ag positive and/or serum HBV DNA positive patients, prophylactic anti-HBV drugs should be used before treatment. If the liver function damage after treatment is caused by the activation of hepatitis B virus, anti-hepatitis B virus drug therapy should be strengthened, and positive symptomatic treatment should be carried out. If grade 1 or 2 liver toxicity occurs, there is no need for adjusting dosage, but if grade 3 or 4 liver toxicity occurs, apatinib dosage should be suspended, and the dosage should be performed with first dosage reduction after toxicity decreasing to grade 1.

6.3.5 Camrelizumab

The adverse events related to camrelizumab may be immunologically related and may occur shortly after the first dose or several months after the last dose. If the situation listed the following table occurs, the administration of camrelizumab should be suspended. If the investigator considers the benefit/risk ratio of the subject during the clinical operation and believes that the operation listed the following table cannot be performed or encounters situations not listed in the following Table, the investigator must suspend or resume the administration of camrelizumab.

The dose adjustment of camrelizumab is performed according to the following principles.

Table 4: Dose adjustment of camrelizumab

Adverse events	Grade	Treatment adjustment
Reactive capillary endothelial proliferation	Grade 3	Suspend drug
	Grade 4	Permanent withdrawal
**Immune-related adverse events**		
Pneumonia	Grade 2	Suspend drug until recover to Grade 0-1
	Grade 3-4 or Recurrent Grade 2	Permanent withdrawal
Diarrhea and colitis	Grade 2-3	Suspend drug until recover to Grade 0-1
	Grade 4	Permanent withdrawal
Hepatitis	Grade 2	Suspend drug until recover to Grade 0-1
	Grade 3-4	Permanent withdrawal
Nephritis	Grade 2-3 elevated creatinine	Suspend drug until recover to Grade 0-1
	Grade 4 elevated creatinine	Permanent withdrawal
Endocrine disease	Grade 2-3	Suspend drug until recover to Grade 0-1
	Grade 4	Permanent withdrawal
Cutaneous AEs	Grade 3	Suspend drug until recover to Grade 0-1
	Grade 4	Permanent withdrawal
Thrombopenia	Grade 3	Suspend drug until recover to Grade 0-1
	Grade 4	Permanent withdrawal
Other irAEs	Grade 3-4 elevated blood amylase or lipase<br>Grade 2-3 pancreatitis<br>Grade 2 myocarditis<br>Grade 2-3 other irAEs for the first time	Suspend drug until recover to Grade 0-1
	Grade 4 pancreatitis or recurrent pancreatitis<br>Grade 3-4 myocarditis<br>Grade 3-4 encephalitis<br>Grade 4 other irAEs for the first time	Permanent withdrawal
Recurrent or persistent AEs	Recurrent Grade 3-4<br>Grade 2 or 3 AEs did not recurrent to grade 0-1 within 12 weeks of the last treatment	Permanent withdrawal
infusion reaction	Grade 2	Slow down the drip rate or suspend the drug

	Grade 3-4	Permanent withdrawal
Note:<br>a. For subjects experiencing intolerable or persistent grade 2 drug-related adverse events, the investigator may suspend camrelizumab as appropriate; if the persistent grade 2 adverse reaction fails to recover to grade 1 or below within 12 weeks after the last dose, the medication should be terminated.<br>b. In the event of any recurrent grade 3 drug-related AE or any life-threatening event, the medication should be terminated.		

7 Concomitant Medication or Treatment

7.1 Application of Antiemetic Drugs

a. Preventive antiemetic therapy: 5-HT3 antagonist given 1 hour before chemotherapy.
b. Delayed vomiting: metoclopramide 20 mg BID and the NK-1 receptor antagonist aprepitant (125 mg, 80 mg, and 80 mg for day 1-3, respectively) were recommended. The 5-HT3 antagonist can replace metoclopramide.

7.2 Application of Granulocyte Colony-stimulating Factor (G-CSF)

The use of G-CSF is not advocated for primary prevention; when white blood cell count (WBC) and/or ANC are significantly reduced, G-CSF treatment can be given according to the judgments of clinicians; if grade 3 or 4 neutropenia or fever (oral temperature > 38.3°C or > 37.8°C lasting for more than one hour) is noted, the following regimens are excepted for dose adjustment. Secondary prevention can also be given; the use of G-CSF should be more than 24 hours away from the end of chemotherapy drugs. Polyethylene glycol-conjugated recombinant human granulocyte stimulating factor is an alternative agent for the prevention of serious neutropenia.

7.3 Combined drug usage and treatment

7.3.1 Permissible Treatment

Supportive treatment, including pain treatment, blood transfusion, intravenous nutrition, etc.

7.3.2 Inadmissible Treatment

During the study period, patients could not accept other experimental drugs and any other form of radiotherapy and chemotherapy, but the follow-up anticancer treatment for patients who fails to follow the treatment plan is not limited.

8 Observation and Assessment During Treatment

The following items need to be assessed during the treatment period:

a) Nasopharynx and neck MRI and nasopharyngeal endoscopy will be performed before and after induce chemotherapy and at the end of radiotherapy, and complete response (CR), partial response (PR), stable disease (SD) or progressive disease (PD) will be evaluated with the Response Evaluation Criteria in Solid Tumors (RECIST) version 1.1. Chest CT and abdominal CT/MR will be reexamined after treatment.

b) General conditions.

c) Acute and late toxicity assessment, including hematological toxicity, gastrointestinal reactions, hepatotoxicity, nephrotoxicity, mucositis, neurotoxicity, ototoxicity, etc.

d) Immune-related toxicity assessment.

e) Laboratory tests: Routine blood tests and blood biochemistry are required on day 0, day 8, and day 15 of chemotherapy for each cycle; the following tests

are routinely suggested but not mandated before each cycle of immunotherapy: thyroid function (TSH, FT3, FT4), coagulation function (APTT, PT, FIB, INR, D-D), serum amylase, lipase, myocardial enzyme profile, hypersensitivity troponin, brain natriuretic peptide, inflammatory cytokine profile, etc.

f) Indirect nasopharyngoscopy will be performed to examine the tumor in the nasopharynx every week, and the regression of enlarged lymph nodes should be observed and measured. Nasal endoscopy will be performed before and after the treatment course and is also required after each cycle of chemotherapy or immunotherapy.

9 Exploratory analysis and translational study

9.1 Exploratory analysis

Pathologic evaluation of endoscopic biopsy and/or lymph nodes biopsy after induction phase would be conducted. The primary efficacy endpoint was DMFS, which was defined as the interval between the initial treatment and distant failure.

EBV DNA clearance rate.

9.2 Translational study

a) Signed informed consent is required prior to each biological sample collection.

b) Tissue specimens from nasopharyngeal and/or neck mass biopsies or fine needle aspiration prior to treatment and after induction therapy, or from recurrence or metastasis site if recurrent or metastasis event occurred.

c) Blood samples, collected at the following time points: before treatment, after induction therapy, the end of radiotherapy, the end of maintain phase, and the time point of distant metastasis or recurrence events.

d) Clinical specimens for the above cases whose informed consent have been

signed will be collected by designated professionals, and all specimens collected will be used for subject-related scientific research only.

e) After collection, tumor tissue specimens will be collected by the principal investigator and stored in a refrigerator at -80 °C or transferred to a designated laboratory for processing and analysis.

f) Hematology specimens are collected, centrifuged, and stored by the principal investigator, or transferred to the designated laboratory for biological sample processing and analysis.

10 Safety Evaluation

10.1 Adverse Event Indicators

Toxicity evaluation should be performed weekly during the treatment and four weeks after the end of treatment. All observations relevant to the safety of the drug under study will be documented in the case report form (CRF) and the final summary report. Safety indicators are as follows: adverse events, laboratory changes (hematology, blood biochemistry), changes in vital signs (blood pressure, heart rate, respiratory rate, body temperature), and changes in electrocardiogram and chest CT.

10.2 Severe Adverse Events (SAEs)

A SAE is any life-threatening medical adverse event that occurs at a variety of drug doses:

a. Causing death or endangering life. It should be noted that the term "life-threatening" in the definition of "severe" means that at the time of the event, the patient is at risk of death. It's not a hypothetical event.
b. Causing permanent or severe disability/dysfunction.
c. Teratogenicity/birth defects.

d. Resulting in prolonged hospital stays.
e. Lead to secondary tumors.
f. Other unpredictable drug related AEs

In the event of a serious adverse event, regardless of whether it is related to the study drug, the investigator should immediately notify the Clinical Research Center (GCP) of the table of serious adverse events within 1 business day (in any case). The form must be dated and signed by the person who filled it

All adverse events, whether or not considered drug related, should be documented on the case report, including diagnosis, start/end time, intervention taken, discontinuation of treatment, salvage measure, outcome, and other possible causes. The investigators should determine the relationship of all adverse events to treatment and the severity of the events. The severity of acute adverse events should be graded according to CTC AE (version 5.0). The most severe severity should be recorded in the CRF.

10.3 Record of Events

All adverse events, whether or not considered drug related, should be documented on the case report, including diagnosis, start/end time, intervention taken, discontinuation of treatment, salvage measure, outcome, and other possible causes. The investigators should determine the relationship of all adverse events to treatment and the severity of the events. The severity of acute adverse events should be graded according to CTC AE (version 5.0). The most severe severity should be recorded in the CRF.

11 Follow Up

After completion of radiotherapy, patients are followed up every 3 months during the first 3 years and every 6 months thereafter until death. The nasopharynx will be

assessed by endoscopy approximately 4 weeks after completion of radiotherapy. Further investigations with MRI or CT will be arranged 3 months after completion of radiotherapy. Other follow-up items include physical examination, blood examination, chest CT or X-ray, abdominal sonography or CT, etc. Treatment responses are also evaluated according to the RECIST, version 1.1. If residual disease is found, whether to treat and which treatment modalities to be employed will be decided by individual clinician. For statistical purpose, any residual disease found 12 weeks after completion of RT will be considered as local failure. Similarly, any residual nodal disease within 12 weeks after RT is considered as regional failure.

Clinical diagnosis is accepted for sites not easily accessible if classical changes are shown on imaging. The dates of diagnosis of local, nodal, and distant failure will be recorded. The earliest date of detecting symptomatic late onset toxicities and the eventual maximum grade by the Late Radiation Morbidity Scoring Criteria of the Radiation Therapy Oncology Group (RTOG) and European Organization for Research and Treatment of Cancer (EORTC) should be recorded. All patients will be followed-up until death and cause of death recorded. Deaths due to unknown cause are counted as death due to NPC if disease is still present at last assessment.

12 Safety Measures and Quality Control

a) Researchers is responsible for obtaining informed consent signed by each subject or his or her agent; carefully fill out the case report form; complete records of laboratory examinations, clinical records, and original medical records of the subjects.

b) Make monitoring plan of adverse effects and emergency plan.

c) Research plan is made by all investigators and approved by Ethics Committee.

d) Develop all kinds of standard operation procedures related to this study.

e) Establish standardized evaluation system to unify diagnostic criteria, curative effect judging criteria, etc.

f) Establish professional statistical plan.

g) Research staffs are trained before the study.

h) Arrange quality controller, make quality control plan and check regularly.

i) Set up coordination committee, curative effect judging group and follow-up team.

13 Data Collection and Data Management

13.1 Case Report Form (CRF)

The CRF is completed by the investigator and must be completed by each enrolled patient. After the completed CRF is reviewed by the clinical supervisor, the first copy is transferred to the data administrator for data entry and management.

13.2 Data Entry and Modification

All information about the enrolled patients after registration will be sent to Sun Yat-Sen University Cancer Center for management. We have stewards taking charge of data entry and management. Data administrators use EpiData 3.1 software to compile data entry program and carry out data entry and management. In order to ensure the accuracy of the data, two data administrators should independently conduct double input and proofreading. If there are any questions in the CRF, the data administrator will send questioned data to the investigator for verification. The investigator should answer and return the questions as soon as possible. The data administrator can modify, confirm and enter the data according to the answers of the investigator.

13.3 Data Locking

After confirming that the established database is accurate, the main researchers, data administrators, and statisticians will lock the data. The locked data file will not be

modified.

14 Statistical Analysis

14.1 Endpoint Definitions

14.1.1 Primary End Point

The primary endpoint was DMFS, which was defined as the time from the initiation of treatment to documented distant metastasis.

14.1.2 Secondary End Points

Response rate. Approximately one week after completing induction therapy, the responses to induction therapy are assessed by imaging by independent image committee. The nasopharynx and cervical nodes should be assessed by endoscopy and MRI or CT at approximately 12 weeks after completion of radiotherapy. Other examinations included chest CT, abdominal CT/MR, and bone scans. Tumor response is classified according to the RECIST criteria, version 1.1. Complete response is defined as the disappearance of all target lesions. Any pathological lymph nodes (whether target or nontarget) must have been reduced in the short axis to <10 mm. Partial response is defined as an at least 30% decrease in the sum of diameters of the target lesions, with the baseline diameter sum serving as the reference. Progressive disease is defined as an at least 20% increase in the sum of diameters of the target lesions, with the smallest sum during study serving as the reference (including the baseline sum). In addition to a relative increase of 20%, the sum must also demonstrate an absolute increase of at least 5 mm. Stable disease is defined as both insufficient size reduction to qualify as partial response and an insufficient increase to be considered progressive disease, with the smallest diameter sum during the study serving as the

reference.

PFS, which is defined as the time from the initiation of induction therapy to documented distant metastasis, locoregional recurrence, or death from any cause, whichever occurred first

OS, which is defined as the time from the initiation of induction therapy to any-cause death.

LRFS, which is defined as the time from the initiation of induction therapy to documented locoregional recurrence.

Treatment toxicity. The incidence of serious toxicity (treatment-related adverse events and immune-related adverse events) during induction phase, radiotherapy phase, and maintain phase, late adverse events, compliance to treatment were included. Adverse events refer to any adverse medical events that occur on the patient. They do not necessarily have a causal relationship with treatment. Investigators should keep a detailed record of any adverse events that occur in the patients. The record of adverse events shall include a description of the adverse events, the time of occurrence, severity, duration, measures taken, and the final outcomes. Investigators should assess the possible association between the adverse events and the tested drugs according to the five-level classification of "positive relevance, possible irrelevance, positive irrelevance, and inability to determine." Treatment-related adverse events are assessed according to NCI-CTCAE version 5.0. Acute toxicities include hematological toxicity, mucositis, allergic reactions and other adverse events and serious adverse events. The severity of immune-related adverse events was graded according to the NCI-CTCAE version 5.0. The severity of late adverse events was graded according to the Radiation Therapy Oncology Group and European Organization for Research and Treatment of Cancer late radiation morbidity scoring scheme.

Quality of life. EORTC QLQ-C30 and QLQ-H&N35 (v1.0) are used to assess life quality of patients, and the change of their life quality is recorded and evaluated weekly from before the beginning of treatment to the last dose of the camrelizumab.

14.2 Determination of the Sample Size

Considering over half distant metastasis events occurred within one year after radical treatment, the primary endpoint of this study was the DMFS at the time point of 1 year. The following set of hypotheses were considered:

H_0: The maximum 1-year DMFS rate of treatment cohort is not more than 76%;

H_A: The minimum 1-year DMFS rate of treatment cohort is not less than 90%.

The historical data estimation is based on the prognostic data of N3 population reported from our center.[13] According to the Fleming single-stage design, the maximum effective rate (P0) was 76%, the minimum effective rate (P1) was 90%, unilateral α was set as 0.05, and the power was set at least 80%, 46 patients should be enrolled. Assuming an 8% dropout rate, we estimated that the study would need to include a total of 50 patients. If 7 of the first 46 evaluable patients failed, the DMFS rate would be considered unacceptable, and the regimen would be declared promising if at least 40 successes of the first 46 evaluable patients were observed.

14.3 Analytical Approach

The therapeutic effects will be analyzed by both full analysis set (intention-to-treat) and per-protocol set. Patients who completed radical radiotherapy and at least one dose of induction therapy were included in the per-protocol analysis set. Safety analysis will be performed in safety population, which is made up of eligible cases that received at least one dose of protocol-defined treatment.

The study results will be reported after universal 1-year follow-up (for the primary endpoint analysis), 2-year and/or 3-year follow-up, and 5-year follow-up (for the long-term results).

15 Ethical Considerations

This study must be approved by an appropriate institutional ethic committee.

An informed consent must be obtained from individual patients. Copy of the Consent Form, contact number of investigator and ethics committee will be available to patient on request.

All serious and unexpected adverse events or death related to the drugs or radiotherapy must be reported to the study coordinator immediately. Serious adverse events (SAE) to be reported include all deaths during or within 30 days of protocol treatment regardless of cause, grade 5 toxicity, life-threatening grade 4 toxicity, and/or unexpected toxicity. The Study Coordinator of respective center should complete form and fax this within 24 hours to the Principal Investigator (Dr. Yan-Qun Xiang, Tel: 020-87343379, Fax: 020-87343379), the center of clinical trials, the institutional ethic committee and Sun Yat-Sen University Cancer Center. Together with the Principal Investigator, appropriate and prompt action will be taken if warranted. Reactions and deaths beyond 30 days from protocol treatment that are judged definitely unrelated to treatment should not be reported.

16 Administration of Experimental Drugs

Jiangsu Hengrui Pharmaceutical provided nab-paclitaxel and apatinib free of charge and camrelizumab free of partial charge. The management, distribution, and recovery of clinical drugs in this trial shall be the responsibility of designated researcher. The researcher must ensure that all trial drugs are used only for subjects participating in the clinical trial, that their doses and usage are in accordance with the trial scheme, and that the remaining drugs are returned to the manufacturer. Experimental drugs should not be transferred to any non-clinical trial participant.

17 Progress of Study

Expected trial start time: May 2020;

Expected completion time: May 2021;

Expected end of trial: May 2026.

18 Principle center and investigator

Principle center: Sun Yat-Sen University Cancer Center

Principle Investigator: Yan-Qun Xiang

Address: Dongfeng East Road 651, Guangzhou 510060

Telephone: 020-87343379

Fax: 020-87343379

19 Bibliography

1. Chen YP, Chan ATC, Le QT, Blanchard P, Sun Y, Ma J. Nasopharyngeal carcinoma. Lancet (London, England) 2019;394:64-80.
2. Huang ZL, Liu S, Wang GN, et al. The prognostic significance of PD-L1 and PD-1 expression in patients with nasopharyngeal carcinoma: a systematic review and meta-analysis. Cancer Cell Int 2019;19:141.
3. Mai HQ, Chen QY, Chen D, et al. Toripalimab or placebo plus chemotherapy as first-line treatment in advanced nasopharyngeal carcinoma: a multicenter randomized phase 3 trial. Nature medicine 2021.
4. Wang FH, Wei XL, Feng J, et al. Efficacy, Safety, and Correlative Biomarkers of Toripalimab in Previously Treated Recurrent or Metastatic Nasopharyngeal Carcinoma: A Phase II Clinical Trial (POLARIS-02). Journal of clinical oncology : official journal of the American Society of Clinical Oncology 2021;39:704-12.
5. Fang W, Yang Y, Ma Y, et al. Camrelizumab (SHR-1210) alone or in combination with gemcitabine plus cisplatin for nasopharyngeal carcinoma: results from two single-arm, phase 1 trials. The Lancet Oncology 2018;19:1338-50.
6. Yang Y, Qu S, Li J, et al. Camrelizumab versus placebo in combination with gemcitabine and cisplatin as first-line treatment for recurrent or metastatic nasopharyngeal carcinoma (CAPTAIN-1st): a multicentre, randomised, double-blind, phase 3 trial. The Lancet Oncology 2021;22:1162-74.
7. Schmittnaegel M, Rigamonti N, Kadioglu E, et al. Dual angiopoietin-2 and VEGFA inhibition elicits antitumor immunity that is enhanced by PD-1 checkpoint blockade. Science translational medicine 2017;9.
8. Allen E, Jabouille A, Rivera LB, et al. Combined antiangiogenic and anti-PD-L1 therapy stimulates tumor immunity through HEV formation. Science translational medicine 2017;9.
9. Xu J, Zhang Y, Jia R, et al. Anti-PD-1 Antibody SHR-1210 Combined with Apatinib for Advanced Hepatocellular Carcinoma, Gastric, or Esophagogastric Junction Cancer: An Open-label, Dose Escalation and Expansion Study. Clinical cancer research : an official journal of the American Association for Cancer Research 2019;25:515-23.
10. Li Q, Wang Y, Jia W, et al. Low-Dose Anti-Angiogenic Therapy Sensitizes Breast Cancer to PD-1 Blockade. Clinical cancer research : an official journal of the American Association for Cancer Research 2020;26:1712-24.
11. Liu J, Liu Q, Li Y, et al. Efficacy and safety of camrelizumab combined with apatinib in advanced triple-negative breast cancer: an open-label phase II trial. J Immunother Cancer 2020;8.
12. Xie L, Xu J, Sun X, et al. Apatinib plus camrelizumab (anti-PD1 therapy, SHR-1210) for advanced osteosarcoma (APFAO) progressing after chemotherapy: a single-arm, open-label, phase 2 trial. J Immunother Cancer 2020;8.
13. Sun X, Su S, Chen C, et al. Long-term outcomes of intensity-modulated

radiotherapy for 868 patients with nasopharyngeal carcinoma: an analysis of survival and treatment toxicities. Radiotherapy and oncology : journal of the European Society for Therapeutic Radiology and Oncology 2014;110:398-403.

Appendix I: The 8th editions of the AJCC Staging System of Nasopharyngeal Carcinoma

**Tumor stage**	
Tx	Primary tumour cannot be assessed
T0	No tumour identified, but there is EBV-positive cervical node(s) involvement
T1	Nasopharynx, oropharynx, or nasal cavity without parapharyngeal extension
T2	Parapharyngeal extension, adjacent soft tissue involvement (medial pterygoid, lateral pterygoid, prevertebral muscles)
T3	Bony structures (skull base, cervical vertebra) and/or paranasal sinuses
T4	Intracranial extension, cranial nerve, hypopharynx, orbit, extensive soft tissue involvement (beyond the lateral surface of the lateral pterygoid muscle, parotid gland)
**Node stage**	
Nx	Regional lymph nodes cannot be assessed
N0	No regional lymph node metastasis
N1	Unilateral cervical, unilateral, or bilateral retropharyngeal lymph nodes, above the caudal border of cricoid cartilage; ≤ 6 cm
N2	Bilateral metastasis in lymph node(s), ≤ 6 cm in greatest dimension, above the caudal border of cricoid cartilage
N3	> 6 cm and/or below caudal border of cricoid cartilage (regardless of laterality)
**Metastasis**	
M0	No distant metastasis
M1	Distant metastasis
**TNM stage**	
I	T1 N0 M0
II	T2 N0–1 M0, T0–1 N1 M0
III	T3 N0–2 M0, T0–2 N2 M0
IVa	T4 or N3 M0
IVb	Any T, any N, M1

Appendix II: ECOG Performance Status and Scores

Score	ECOG Performance status*
0	Fully active, able to carry on all pre-disease performance without restriction
1	Restricted in physically strenuous activity but ambulatory and able to carry out work of a light or sedentary nature, e.g., light housework, office work
2	Ambulatory and capable of all selfcare but unable to carry out any work activities; up and about more than 50% of waking hours
3	Capable of only limited selfcare; confined to bed or chair more than 50% of waking hours
4	Completely disabled; cannot carry on any selfcare; totally confined to bed or chair
5	Dead
* Oken M, Creech R, Tormey D, et al. Toxicity and response criteria of the Eastern Cooperative Oncology Group. Am J Clin Oncol. 1982;5:649-655.<br>Note: ECOG denotes Eastern Cooperative Oncology Group	

Appendix III: Acute Induction Chemotherapy and Chemoradiotherapy Toxicity Graded by Common Terminology Criteria for Adverse Events (CTCAE 5.0).

The CTCAE v5.0 manual can be found at the following URL: https://evs.nci.nih.gov/ftp1/CTCAE/CTCAE_5.0/

Appendix IV: Late Onset Toxicities Graded by RTOG/EORTC System

RTOG/EORTC System manual can be found at the following URL: https://www.eortc.org/rog-guidelines/

Efficacy and Safety of Camrelizumab and Apatinib Combined with Induction Chemotherapy and Concurrent Chemoradiotherapy for Stage TanyN3M0 Nasopharyngeal Carcinoma: A Phase II QUINTUPLED Trial

Final protocol (Second version: March 20, 2021)
Trial Code: QUINTUPLED

**Principal Investigator**	Dr. Yan-Qun Xiang<br>Department of Nasopharyngeal Carcinoma<br>Cancer Center, Sun Yat-Sen University<br>651 Dongfeng Road East, Guangzhou, China<br>Tel: (020)-87343379; Fax: (020)-87343379<br>E-mail: xiangyq@mail.sysu.edu.cn
**Participating Center**	
Sun Yat-Sen University Cancer Center, Guangzhou, China	
Lead investigators	Dr. Yan-Qun Xiang<br>Dr. Chong Zhao<br>Department of Nasopharyngeal Carcinoma<br>Dr. Changqing Xie<br>Thoracic and GI Malignancies Branch<br>National Cancer Institute, NIH
**Data Manager & Statistician**	Dr. Qing Liu<br>Department of Medical Statistics and Epidemiology, Cancer Center, Sun Yat-Sen University<br>Dr. Yan-Fang Ye<br>Clinical Research Design Division, Sun Yat-Sen Memorial Hospital, Sun Yat-Sen University
**Honorary Adviser**	Dr. Xiang Guo<br>Dr. Hai-Qiang Mai<br>Department of Nasopharyngeal Carcinoma<br>Cancer Center, Sun Yat-Sen University

Activation Date

First approved version: April 25, 2020

Current Edition: March 20, 2021

1 Background

Nasopharyngeal carcinoma (NPC) differs from other head and neck cancers with unique geographical, etiological, and biological features.[1] It is endemic in the area of Southern China, Southeast Asia, and North Africa. More than 70% of NPC patients are classified as having locoregionally advanced disease among the 129,000 newly diagnosed cases annually.[1] Induction chemotherapy followed by concurrent chemoradiotherapy is the current standard treatment for locoregionally advanced NPC, recommended by the National Comprehensive Cancer Network (NCCN) Guidelines as level 1 evidence. With the application of MRI, intensity-modulated radiotherapy and combined chemoradiotherapy has substantially improved locoregional control. However, distant metastasis predominates as the pattern of treatment failure. In particular, approximately 50% of patients with T_{any}N3M0 diseases eventually develop distant metastasis after undergoing abovementioned radical treatment. Therefore, more effective treatment strategies are needed for this patient population.

Due to the properties of NPC with the abundant lymphocytic infiltrations and elevated expressions of programmed death-ligand 1 (PD-L1)[2], immune checkpoint blockade therapy has considerable therapeutic potential for patients with NPC. Its effect on the treatment has confirmed in recurrent or metastatic NPC (RM-NPC) in the first-, second-, and greater-line settings.[3-6] However, there are no available reports assessing the value of adding immunotherapy to standard treatment in the curative setting to date.

Camrelizumab (SHR-1210) is a fully humanized immunoglobulin G4/k PD-1 monoclonal antibody, which is well tolerated with positive antitumor activity in RM-NPC. At the same time, antiangiogenic therapies have been shown to sensitize anti-PD-1/PD-L1 treatment by increasing PD-L1 expression and CD8+ T cell infiltration in preclinical models.[7,8] Remarkably, apatinib, an orally administered vascular endothelial growth factor receptor 2 (VEGFR2) tyrosine kinase inhibitor, has demonstrated antitumoral activity and synergetic effects with camrelizumab in several solid tumors.[9-12] Therefore, we evaluated the effect and safety of the combination of camrelizumab

and apatinib with standard treatment regimen as the curative setting in stage $T_{any}N3M0$ NPC.

2 Objectives

2.1 Primary Objectives

To assess distant metastasis-free survival of camrelizumab plus apatinib combined with induction chemotherapy (TPC; NAB-paclitaxel, cisplatin and capecitabine) and concurrent chemoradiotherapy for high-risk of metastatic NPC patients (stage $T_{any}N3M0$).

2.2 Secondary Objectives

To evaluate response rate, overall survival (OS), failure-free survival (FFS) rate, locoregional recurrence-free survival (LRFS), toxicity profile, treatment adherence of camrelizumab plus apatinib combined with induction chemotherapy (TPC; NAB-paclitaxel, cisplatin and capecitabine) and concurrent chemoradiotherapy for high-risk of metastatic NPC patients (stage $T_{any}N3M0$).

3 Trial Scheme

Nasopharyngeal carcinoma
Histology: non-keratinizing
Staging: TanyN3M0 by AJCC 8th system*

↓

Induction phase
3 cycles of Camrelizumab and Apatinib combined with induction chemotherapy (TPC; NAB-paclitaxel, cisplatin and capecitabine)

↓

Concurrent chemoradiotherapy phase
Intensity-modulated radiotherapy with 2 cycles of concurrent cisplatin and Camrelizumab every 3 weeks

↓

Maintenance phase
Camrelizumab every 3 weeks up to 1 year

↓

Primary endpoint: Distant metastasis-free survival
Secondary endpoint: Treatment response, toxicity profile, overall survival, failure-free survival, local relapse-free survival, treatment adherence

*The 8th edition of the American Joint Commission on Cancer staging system.

4 Subject Enrollment

4.1 Eligibility Criteria

a) Patients newly diagnosed with histologically confirmed differentiated or undifferentiated non-keratinized carcinomas (according to the world health organization [WHO] histologically type).

b) Patients with tumors staged as $T_{any}N3M0$ (as defined by the 8th AJCC edition, appendix I).

c) No sign of distant metastasis.

d) Without previous anticancer treatment.

e) Without a history of other malignancies.

f) Age between 18-65 years.

g) Adequate bone marrow function: White blood cell count of $\geq 4.0\times10^9$/L, absolute neutrophil count (ANC) of $\geq 2.0\times10^9$/L, platelet count of $\geq 100\times10^9$/L, and hemoglobin concentrations of $\geq$ 90 g/L.

h) Sufficient renal function: creatinine clearance rate at greater than or equal to 60 mL/min.

i) Normal liver function: aspartate aminotransferase (AST) and alanine aminotransferase (ALT) $\leq$ 2.0 times the upper limit of normal (ULN).

j) Eastern Cooperative Oncology Group performance status of 0 or 1.

k) Signed informed consent form.

4.2 Exclusion Criteria

a) Patients suffering from basaloid squamous cell carcinoma or WHO Type keratinizing squamous cell carcinoma.

b) Previous anticancer treatment, including chemotherapy, radiotherapy, and surgery (except diagnostic).

c) The presence of distant metastasis at initial diagnosis.

d) Patients who are pregnant or lactating (in women of child-bearing age, pregnancy test should be considered and effective contraception during the treatment period should be emphasized).

e) Patients suffering from any severe uncontrollable infections, serious physical or mental diseases or abnormal laboratory tests, which may incur unacceptable risk, negatively affect trial compliance, or affect the administration, distribution, metabolism, and excretion of study drugs. For example, unstable cardiac disease necessitating treatment, chronic hepatitis renal disease, poorly controlled diabetes (fasting plasma glucose greater 1.5 × the ULN), emotional disturbance, mental disorders, abnormal central nervous system, chronic diarrhea, ascites, and pleural effusion.

f) Hypertension, and cannot be well controlled by antihypertensive drugs (systolic blood pressure > 140 mmHg or diastolic blood pressure > 90 mmHg).

g) Human immunodeficiency virus (HIV) infection or known acquired immunodeficiency syndrome (AIDS), untreated active hepatitis (hepatitis B is defined as HBV-DNA ≥ 500 IU/ml; hepatitis C is defined as HCV-RNA higher than the detection limit of the analytical method) or co-infection of hepatitis B and C.

h) Patients who cannot cooperate with routine follow-up because of psychological, social, family, or geographical reasons.

i) Patients with a history of other malignancies before admission.

j) Patients with a history of psychotropic substance abuse or drug use.

k) Receive experimental treatment from other clinical studies at the same time (during the treatment period of this trial).

l) Refuse to sign the informed consent form.

m) Long-term use of immunosuppressive agents after organ transplantation.

4.3 Criteria for withdrawal from protocol treatment

The reasons for subjects' withdraw from the study may include:

a) Evidence of disease recurrence.
b) The patient may withdraw from the study and reject further follow-up at any time for any reason. The reason should be recorded.
c) Unacceptable toxicity, the reasons for which must be recorded.
d) The test results of the subject's β-HCG test indicated that the subject was pregnant. At the same time, pregnancy will also be reported as a serious adverse event reporting procedure.
e) Use of prohibited medications or other medications that the investigator believes will cause a toxic reaction or affect the results of the study.
f) Patients suffering from an intercurrent disease that significantly affects the assessment of clinical status or necessitates discontinuation of the drug, or both.
g) Investigators believe that the termination of treatment is beneficial to patients according to their physical condition.
h) Death.

5 Treatment Plan

5.1 Pretreatment Assessment

All patients are under standardized management for NPC, and they need to perform a series of examinations as well as provide relevant information to confirm pathologic diagnosis and clinical stage before admitted into trial:

a) Signed informed consent.
b) Medical history review.
c) Personal data collection.
d) Current medications and treatment.
e) Body examinations, include height, weight and vital signs.
f) Physical examination of head and neck region, include nasopharynx and cervical lymph nodes.
g) Physical examination of the nervous system.

h) Nasal endoscopy and lesion biopsy.

i) Complete blood count and differential, electrolytes and liver function test.

j) Urinalysis.

k) EBV antibodies and EBV DNA testing.

l) Virological examination (completed within 14 days prior to the first dose): HBsAg (if positive, HBV-DNA is required), HBsAb, HBeAg, HBeAb, HBcAb, HCV-Ab (if positive, HCV-RNA is required) , HIV-Ab.

m) Thyroid function.

n) Coagulation function.

o) Electrocardiogram and Echocardiography.

p) Imaging, including enhanced MRI or enhanced CT of the head and neck (CT is indicated only in patients with contraindication to MRI).

q) Chest CT.

r) ECT bone scan.

s) Abdominal CT/MR.

t) PET/CT is optional and is performed at the discretion of the attending physician.

u) Pregnancy test (applicable to women of childbearing age).

5.2 Pretreatment Staging Criteria

The disease stage of subjects will be staged according to the 8th edition of the Union for International Cancer Control / American Joint Committee on Cancer (UICC/AJCC) staging system (Appendix I).

5.3 Chemotherapy, target and immunotherapy

5.3.1 Induction therapy phase

1. Camrelizumab

Camrelizumab was intravenously given at dose of 200 mg, administered as a 30-minutes (no less than 20 minutes, no more than 60 minutes) infusion on day 1. In

particular, an actual dose of 3 mg/kg was given to those weighing less than 40 kg. Camrelizumab will be administrated throughout the trial every three weeks up to one year.

2. Induction chemotherapy with TPC regimen

The TPC regimen consisted of NAB-paclitaxel at a dose of 200 mg per square meter, administered over 30 minutes infusion on day 1, followed by cisplatin at a dose of 60 mg per square meter on day 1; No premedication against allergic reaction was given before the NAB-paclitaxel; And capecitabine at a dose of 1000 mg per square meter, taken orally twice a day on days 1 to 14; repeat every 21 days for 3 cycles.

After the infusion of camrelizumab, NAB-paclitaxel and cisplatin are successively administered intravenously after an interval of at least 30 min.

3. Apatinib

Apatinib taken orally at a dose of 250 mg daily on days 1 to 5 per week for a total of 8 weeks. The drug should be administered on the first day of initial treatment and discontinued one week before radiotherapy.

5.3.2 Concurrent chemotherapy with cisplatin

Concurrent chemotherapy with cisplatin

The concurrent chemotherapy consisted of cisplatin at a dose of 100 mg per square meter, administered as a 4-hour infusion; repeat every 21 days for 2 cycles.

Cisplatin administration follows local hydration protocols to prevent/minimize nephrotoxicity. Pre-hydration (at least 12 hours before cisplatin administration, D0) and post-hydration (D1-3) with over 2000 ml of normal saline infused each time are both needed. The use of mannitol (D1) and furosemide (D1) is essential for it helps to reduce cisplatin-related nephrotoxicity by diuresis. Twenty-four hours urinary output should be recorded (D0-D3) and urinalysis will be performed (D2-D3). Electrolytes and serum creatinine are monitored during treatment and a follow-up creatinine within 24-48 hours of the cisplatin dose is recommended. One hour before concurrent chemotherapy, patients are given 5-hydroxytryptamine-3 receptor antagonists for anti-nausea (D1).

The NK-1 receptor antagonist aprepitant QD were recommended in D1-D3.

5.4 Radiotherapy

5.4.1 Radiation Therapy

Radiation therapy with intensity-modulated radiotherapy (IMRT) techniques is mandatory. Target volumes are defined following the International Commission on Radiation Units and Measurements (ICRU) reports 50 and 62. The principle of target volume determination for IMRT and prescribed dose and fractionation areas are described as follows.

5.4.2 Target Volume Determination for IMRT

Gross tumor volume (GTV)

(1) Gross tumor target volume of primary nasopharyngeal focus (GTVnx): primary nasopharyngeal tumors and their invasive areas revealed by clinical examination and imaging examination.

(2) Clinically involved gross cervical lymph nodes (GTVnd): cervical metastatic lymph nodes clinically affected and/or imaged (imaging diagnostic criteria: a. short diameter >1 cm; b. necrotic focus in the center; c. extracapsular invasion; d. clustered or clustered short diameter >0.8 cm).

Clinical target volume (CTV)

(3) Nasopharyngeal clinical target volume I (CTV1, nasopharyngeal high-risk area): GTVnx expands 0.5-1.0 cm forward, upper, lower, and bilateral, and 0.2-0.3 cm backward (according to the characteristics of adjacent tissue structure), which should also include all nasopharyngeal mucosa and 0.5 cm below.

(4) Clinical target volume II (CTV2, nasopharyngeal low-risk area, cervical metastatic lymph node area, and cervical area requiring preventive irradiation): CTV1 expands 0.5-1.0 cm forward, upper, lower, and bilateral, 0.2-0.3 cm backward (according to adjacent tissue structure characteristics) including the

lymphatic drainage area of GTVnd and extends to negative lymph node drainage area requiring preventive irradiation (Table 1).

Table 1. Cervical preventive exposure areas

Lymph node metastasis	Cervical lymph node area (CTV2 neck) requiring radiation prevention
II	Ipsilateral II, III, IV, Va, Vb
III	Ipsilateral II, III, IV, Va, Vb
Va	Ipsilateral II, III, IV, Va, Vb
IV	Ipsilateral II, III, IV, Va, Vb, supraclavicular region
Vb	Ipsilateral II, III, IV, Va, Vb, supraclavicular region
Unilateral neck	Contralateral zones II, III, Va

Note: Indications of area are included in CTV2: (1) metastatic lymph nodes in area IIa (> 3 cm); (2) extracapsular invasion of metastatic lymph nodes in area IIa; (3) the metastatic lymph nodes in area IIa push the submandibular gland or have no clear demarcation with the submandibular gland; (4) lymph node metastasis in multiple areas of the ipsilateral whole neck (>4 areas).

Planned target volume (PTV)

PTVnx, PTVnd, PTV1, and PTV2 are GTVnx, GTVnd, CTV1, and CTV2, respectively, with expansion to a certain distance, generally 0.3 cm in the forward, upper, lower, left and right directions, and 0.1-0.2 cm in the backward direction.

5.4.3 Prescribed Dose and Fractionation

All patients will be treated with IMRT using simultaneously integrated boost, 5 fractions per week. The prescribed dose is PTVnx: 68-72 Gy/30-33F, PTVnd: 64-68 Gy/30-33F, PTV1: 60-64 Gy/30-33F, PTV2: 54-58 Gy/30-33F, for the PTVs derived from GTVnx, GTVnd, CTV1, and CTV2, respectively.

5.4.4 Normal Tissue Dose Constraints

Table 2. Normal tissue dose constraint structure.

Structure	Dose constraints
**Critical organ at risk**	
Brain stem	Dmax ≤ 54 Gy
PRV-Brain stem	D1 ≤ 60 Gy
Spinal cord Dmax*	Dmax* ≤ 45 Gy
PRV-Spinal cord	D1†≤ 50 Gy
Optic nerve	Dmax ≤ 54 Gy
PRV-Optic nerve	D1 ≤ 60 Gy
Optic chiasm	Dmax ≤ 54 Gy
PRV-Optic chiasm	D1 ≤ 60 Gy
Temporal lobe	Dmax ≤ 60 Gy
PRV-Temporal lobe	D1 ≤ 65 Gy
**Intermediate-risk organ at risk**	
Pituitary	Dmax < 60 Gy
Mandibular	Dmax < 70 Gy
Temporomandibular Joint	Dmax < 70 Gy
Lens	Dmean‡ < 8 Gy
Eyes	Dmean < 35 Gy
**Low-risk organ at risk**	
Parotid	Dmean < 26 Gy
Parotid	V30¶ < 50%
Cochlea	Dmean < 50 Gy
Tongue	Dmean < 45 Gy
Larynx	Dmean < 45 Gy
PRV = planning organ at risk volume.<br>* Maximum point dose to the target volume.<br>† Dose received by 1% of the target volume.<br>‡ Mean dose to the target volume.<br>¶ At least 50% of the gland will receive <30 Gy (should be achieved in at least one gland)	

5.4.5 Radiotherapy Adjustments for Non-hematological Toxicity

Side effects of radiotherapy may include anorexia, mucositis and skin reaction. The investigator will manage these conditions according to clinical practice. No radiotherapy dose modifications are allowed. Treatment interruptions are allowed if symptomatic mucositis or skin reactions occur that, in the judgment of the attending clinician, warrants a break. The treatment is completed according to protocol for treatment breaks up to and including 14 days. If the break exceeded 14 days, the patient

is removed from protocol treatment, completing treatment at the discretion of his or her physician but followed up and included in the analysis.

5.4.6 Radiotherapy Adjustments for Hematological Toxicity

Radiotherapy will be withheld until ANC > 0.5×10^9/L and platelet > 25×10^9/L.

5.5 Salvage Therapy

Nasopharyngeal surgery, neck dissections, secondary radiotherapy, chemotherapy, or immunotherapy may be given to patients with relapse or metastasis after treatment according to guidelines of National Comprehensive Cancer Network (NCCN).

6 Dosage Adjustment Principle for chemotherapy

6.1 General principle for dosage adjustment

(1) Assessment and grading for toxicity in patients will be carried out using the the Common Terminology Criteria for Adverse Events, version 5.0, of the National Cancer Institute (NCI CTCAE V5.0).

(2) There will be no dose escalation of NAB-paclitaxel, cisplatin and capecitabine (TPC) regimen.

(3) Chemotherapy dosage modifications based on nadir counts and interim nonhematological toxicities from the preceding cycle.

(4) Dosage may not be reduced or discontinued if the investigator considers that the adverse reaction is unlikely to develop into a serious or life-threatening events and will not result in delay or interruption of treatment. There is no need to reduce the dosage or discontinue treatment if anemia can be well alleviated by blood transfusion.

(5) A delay of up to 3 weeks for a cycle will be permitted to allow for the severity of toxic events of grade 3/4 to be reduced to grade 1 or less (except for alopecia, fatigue, malaise, and nail changes). Delays in excess of 3 weeks will necessitate require discontinuation of chemotherapy unless continued therapy is deemed necessary by the investigator.

(6) Chemotherapy must be withheld until the neutrophil count is > 1.5×10^9/L and the platelet count is > 100×10^9/L.

(7) Missing capecitabine doses due to toxicity should be no longer replenished or resumed, and the patient continued the scheduled course of treatment instead.

(8) If creatinine clearance was less than 70 ml/min but greater than 50 ml/min at any time during the previous chemotherapy course. The dose of cisplatin should be reduced by 50% during the next cycle of chemotherapy. If the creatinine clearance rate was less than 50 ml/min during the previous chemotherapy course, subjects should discontinue study therapy.

6.2 Dosage adjustment due to hematologic adverse events

The hematological toxicity is assessed according to the NCI CTC AE V5.0 classification standard, and the dosage adjustment of NAB-paclitaxel and cisplatin is performed according to the following principles.

Dosage adjustment of NAB-paclitaxel and cisplatin for hematologic toxicity[a]				
Toxicity	Grade	Time	NAB-paclitaxel	Cisplatin
Anemia	Any	Any	Full dosage	Full dosage
Neutropenia[b]	Grade 3	Any	Full dosage	Full dosage
	Grade 4	First time	160 mg/m^2	50 mg/m^2
		Second time	120 mg/m^2	40 mg/m^2
		Third time	Stop	Stop
Neutropenic fever[c]	Grade 3-4	First time	160 mg/m^2	50 mg/m^2

		Second time	120 mg/m^2	40 mg/m^2
		Third time	Stop	Stop
Thrombocytopenia	Grade 4	First time	160 mg/m^2	50 mg/m^2
		Second time	120 mg/m^2	40 mg/m^2
		Third time	Stop	Stop

Note:

a. Grade 3 neutropenia or thrombocytopenia, which does not result in prolonged chemotherapy interval, does not require dosage reduction. If this leads to prolonged chemotherapy interval, G-CSF or platelet-enhancing drug support may be considered for the next course of treatment. If this is still not resolved, the dosage should be reduced with the reference to the corresponding grade 4.

b. Grade 3 or 4 neutropenia may be consideration for prevention with G-CSF support during the next course of treatment.

c. Grade 3 or 4 neutropenic fever may be prevented with G-CSF support while the dosage is reduced during the next cycle of treatment. The definition of neutropenic fever: ANC < 1.0×10^9/L with fever ≥ 38.5 ℃ for grade 3 and ANC < 1.0×10^9/L with fever ≥ 38.5 ℃ with life-threatening sepsis for grade 4.

6.3 Dosage adjustment due to non-hematologic adverse events

6.3.1 NAB-paclitaxel

Peripheral neuritis and extremities joint myalgia

If a patient develops one of the above symptoms, the following methods are recommended to adjust the dosage of NAB-paclitaxel according to the duration and severity of the symptoms. Symptomatic treatment with non-steroidal anti-inflammatory drugs can be given alleviate extremities joint myalgia.

Dose adjustment of NAB-paclitaxel for non-hematologic toxicity

Adverse events	Grade	Duration of adverse events		
		1-7 days	Over 7 days	Persistent during treatment
Pain/paresthesia/sensory disturbances, but does not affect function	1	Full dose	Full dose	160 mg/m^2
Pain/paresthesia/sensory disturbances that affect function but do not affect daily activities	2	Full dose	Full dose	120 mg/m^2
Pain/paresthesia/sensory disturbances, with functional impairment, and interference with daily activities	3	Full dose	120 mg/m^2	Stop
Pain/paresthesia/sensory disturbances resulting in loss of function or life threating	4	Stop	Stop	Stop

Abnormal bilirubin and impaired liver function

For HBs-Ag positive or serum HBV DNA positive patients, prophylactic anti-HBV drugs should be used before treatment. If the liver function damage after chemotherapy is caused by the activation of hepatitis B virus, anti-hepatitis B virus drug therapy should be strengthened, and positive symptomatic treatment should be carried out. If grade 2 liver toxicity occurs, there is no need for adjusting NAB-paclitaxel dosage, but if grade 3 liver toxicity occurs, NAB-paclitaxel dosage should be reduced by 20%. Whenever grade 4 liver toxicity occurs, NAB-paclitaxel should be permanently discontinued. If grade 2-4 toxicity occurs, medication will be suspended until the toxicity returns to grade 1 or below before restarting treatment.

Other toxicity

If other grade 3 or less toxic reactions occur, symptomatic treatment should be given as far as possible, with no reduction of NAB-paclitaxel for the next course of treatment. If grade 3 toxicity occurs, when the toxicity reverts from grade 3 to grad 1 or below, the dosage of NAB-paclitaxel for the next course of treatment is reduced by 20%. If grade 4 toxicity occurs, treatment with NAB-paclitaxel should be permanently

discontinued. However, in the event of certain grade 3 or higher adverse events (i.e., fatigue, changes in sexual function, and dry skin without clinical significance), the investigator may continue the treatment at her or his discretion.

6.3.2 Cisplatin

Other non-hematological toxicity

If other toxicity reaction less than grade 3 occur, symptomatic treatment should be given whenever possible. If grade 3 or 4 toxicity occurs, treatment should be suspended, and when toxicity reverts to grade 1 or below, cisplatin should be administered at 80% of the initial dosage for the next course of treatment.

6.3.3 Capecitabin

Once the dosage of capecitabin has been reduced, it should not be increased later. Dosage adjustment is not recommended when grade 1 adverse events occur. Capecitabin treatment should be suspended if grade 2 or 3 adverse events occur. Treatment can be restarted with the original dosage of capecitabin or at the dosage adjusted in the following principles once the adverse reaction has disappeared or the severity has reduced to grade 1. If grade 4 adverse reactions occur, treatment should be suspended until the adverse reactions disappear or the severity is reduced to grade 1, and then treatment should be restarted at 50% of the original dosage. Missing capecitabine doses due to toxicity should be no longer replenished or resumed, and the patient continued the scheduled course of treatment instead.

The detailed dose adjustment of capecitabine is performed according to the following principles.

Table 3: Dose adjustment of capecitabine.

**NCI assessed advent events**	**During treatment**	**Dose of the next cycle (% original dose)**
**Grade 1**	Full dose	Full dose
**Grade 2**		

First time	Suspended until returned to grade 0~1	100%
Second time	Suspended until returned to grade 0~1	75%
Third time	Suspended until returned to grade 0~1	50%
Fourth time	Permanent termination	
**Grade 3**		
First time	Suspended until returned to grade 0~1	75%
Second time	Suspended until returned to grade 0~1	50%
Third time	Permanent termination	
**Grade 4**		
First time	Permanent termination or suspended until returned to grade 0~1 if researcher believe it is in the patient's interest to continue treatment	50%
Note: patients with hand-foot syndrome would be recommended treated with topical emollient, topical corticosteroid cream, or creams containing urea.		

6.3.4 Apatinib

Adverse reactions should be closely monitored during treatment and the dose should be adjusted as needed to enable patients to tolerate the treatment. Once the dose of the drug has been reduced, it should not be increased later. Apatinib 250 mg is recommend orally administrated daily, 5 days every week. In case of adverse reactions related to apatinib, dose frequency should be adjusted according to the following conditions: The recommended frequency should be reduced to:

- 250 mg every two days for the first adjustment;
- Suspended if the relative adverse events occurred second time; After the drug reaction disappeared, the dose before the adjustment could be restored.
- If the patient is still intolerant, stop apatinib permanently.

Hypertension

For patients with hypertension, antihypertensive drugs should be used reasonably before the initiation of apatinib administrated, and blood pressure should be closely monitored during treatment. For patients with grade 1 or 2 hypertension (120-159 mmHg systolic pressure or 80-99 mmHg diastolic pressure) during treatment, there is no need to adjust the dose of apatinib; For grade 3 hypertension (systolic pressure ≥ 160mmHg or diastolic pressure ≥ 100mmHg), apatinib should be stopped. After drug management intervention, apatinib can be continued with reduced dosage if blood pressure is well controlled. Patients with grade 4 hypertension, such as hypertensive crisis and other malignant hypertensive reactions, need to stop taking apatinib immediately and permanently.

Proteinuria

Urine protein should be monitored closely during treatment. Urine routines and/or 24-hour proteinuria are recommended every 2 weeks for the first 2 months and every 4 weeks thereafter. There is no need to adjust the dose of apatinib for grade 1 or 2 urine protein during the treatment. Apatinib should be stopped for grade 3 (24-hour urine protein quantification ≥ 3.5 g), and drug intervention should be performed. After proteinuria is restored to grade 2 or below, apatinib can be continued with reduced dosage. If grade 3 proteinuria occurs again after twice reduction, apatinib should be stopped permanently.

Hand and foot skin reaction

It is recommended that the hands and feet skin reaction drug prevention intervention can be applied with urea ointment, moisturizing cream or aloe gel on the skin of hands and feet from the first day of taking apatinib. For grade 1 reaction, local symptomatic support is recommended without dose adjustment; For grade 2 reaction, dose can be adjusted appropriately; For grade 3 reaction, apatinib should be suspended; After symptomatic support, apatinib can be continued with reduced dosage; if the reaction persist or aggravate, apatinib should be discontinued.

Bleeding or thrombosis

For patients with symptoms of any-reason nosebleed, apatinib should be suspended until the bleeding disappears and apatinib can be continued without dose

adjustment. For grade 2 venous thrombosis, symptomatic treatment and close monitoring was recommended without dose adjustment; For grade 3 or asymptomatic grade 4 reaction, apatinib should be stopped and treated with anticoagulation for at least one week. When the symptoms of thrombosis improve and grade 3-4 bleeding does not occur, apatinib can be continued with adjusted dose, otherwise the drug should be stopped.

Gastrointestinal reaction

For patients with grade 1 reaction, local symptomatic support is recommended without dose adjustment. For grade 2 reaction, especially diarrhea, vomiting and other symptoms, apatinib should be suspended if symptoms did not relieve after symptomatic treatment; For grade 3-4 reaction, apatinib should be discontinued immediately.

Impaired liver function

For HBs-Ag positive and/or serum HBV DNA positive patients, prophylactic anti-HBV drugs should be used before treatment. If the liver function damage after treatment is caused by the activation of hepatitis B virus, anti-hepatitis B virus drug therapy should be strengthened, and positive symptomatic treatment should be carried out. If grade 1 or 2 liver toxicity occurs, there is no need for adjusting dosage, but if grade 3 or 4 liver toxicity occurs, apatinib dosage should be suspended, and the dosage should be performed with first dosage reduction after toxicity decreasing to grade 1.

6.3.5 Camrelizumab

The adverse events related to camrelizumab may be immunologically related and may occur shortly after the first dose or several months after the last dose. If the situation listed the following table occurs, the administration of camrelizumab should be suspended. If the investigator considers the benefit/risk ratio of the subject during the clinical operation and believes that the operation listed the following table cannot be performed or encounters situations not listed in the following Table, the investigator must suspend or resume the administration of camrelizumab.

The dose adjustment of camrelizumab is performed according to the following

principles.

Table 4: Dose adjustment of camrelizumab		
**Adverse events**	**Grade**	**Treatment adjustment**
Reactive capillary endothelial proliferation*	Grade 3	Suspend drug
	Grade 4	Permanent withdrawal
**Immune-related adverse events**		
Pneumonia	Grade 2	Suspend drug until recover to Grade 0-1
	Grade 3-4 or Recurrent Grade 2	Permanent withdrawal
Diarrhea and colitis	Grade 2-3	Suspend drug until recover to Grade 0-1
	Grade 4	Permanent withdrawal
Hepatitis	Grade 2	Suspend drug until recover to Grade 0-1
	Grade 3-4	Permanent withdrawal
Nephritis	Grade 2-3 elevated creatinine	Suspend drug until recover to Grade 0-1
	Grade 4 elevated creatinine	Permanent withdrawal
Endocrine disease	Grade 2-3	Suspend drug until recover to Grade 0-1
	Grade 4	Permanent withdrawal
Cutaneous AEs	Grade 3	Suspend drug until recover to Grade 0-1
	Grade 4†	Permanent withdrawal
Thrombopenia	Grade 3	Suspend drug until recover to Grade 0-1
	Grade 4	Permanent withdrawal
Other irAEs	Grade 3-4 elevated blood amylase or lipase<br>Grade 2-3 pancreatitis<br>Grade 2 myocarditis<br>Grade 2-3 other irAEs for the first time	Suspend drug until recover to Grade 0-1
	Grade 4 pancreatitis or recurrent pancreatitis<br>Grade 3-4 myocarditis<br>Grade 3-4 encephalitis<br>Grade 4 other irAEs for the first time	Permanent withdrawal
Recurrent or persistent AEs	Recurrent Grade 3-4<br>Grade 2 or 3 AEs did not recurrent to grade 0-1 within 12 weeks of the	Permanent withdrawal

	last treatment	
infusion reaction	Grade 2	Slow down the drip rate or suspend the drug
	Grade 3-4	Permanent withdrawal
Note:<br>* For patients with reactive capillary endothelial proliferation in mucous membrane-related sites such as the oral cavity or nasal cavity that cause bleeding symptoms, symptomatic support should be provided and camrelizumab should be suspended and resumed when the aforementioned reactive capillary endothelial proliferation in mucous membrane sites has resolved or when no risk of potential major bleeding (>100 ml) has been assessed by the investigators.<br>† Considering the diversity of causes of rash reactions during combination drug administration, the established treatment regimen was maintained for patients who experienced grade 4 rash reactions that subsided after one week using symptomatic treatment. Patients whose rash reactions persisted for more than two weeks without complete remission, camrelizumab was suspended and chemotherapy drugs continued; and a second cautious attempt may be considered after complete resolution of symptoms evaluated by the investigators and after full communication with the patient.<br>a. For subjects experiencing intolerable or persistent grade 2 drug-related adverse events, the investigator may suspend camrelizumab as appropriate; if the persistent grade 2 adverse reaction fails to recover to grade 1 or below within 12 weeks after the last dose, the medication should be terminated.<br>b. In the event of any recurrent grade 3 drug-related AE or any life-threatening event, the medication should be terminated.		

7 Concomitant Medication or Treatment

7.1 Application of Antiemetic Drugs

a. Preventive antiemetic therapy: 5-HT3 antagonist given 1 hour before chemotherapy.
b. Delayed vomiting: metoclopramide 20 mg BID and the NK-1 receptor antagonist aprepitant (125 mg, 80 mg, and 80 mg for day 1-3, respectively) were recommended. The 5-HT3 antagonist can replace metoclopramide.

7.2 Application of Granulocyte Colony-stimulating Factor (G-CSF)

The use of G-CSF is not advocated for primary prevention; when white blood cell count (WBC) and/or ANC are significantly reduced, G-CSF treatment can be given according to the judgments of clinicians; if grade 3 or 4 neutropenia or fever (oral temperature > 38.3°C or > 37.8°C lasting for more than one hour) is noted, the following regimens are excepted for dose adjustment. Secondary prevention can also be given; the use of G-CSF should be more than 24 hours away from the end of chemotherapy drugs. Polyethylene glycol-conjugated recombinant human granulocyte stimulating factor is an alternative agent for the prevention of serious neutropenia.

7.3 Combined drug usage and treatment

7.3.1 Permissible Treatment

Supportive treatment, including pain treatment, blood transfusion, intravenous nutrition, etc.

7.3.2 Inadmissible Treatment

During the study period, patients could not accept other experimental drugs and any other form of radiotherapy and chemotherapy, but the follow-up anticancer treatment for patients who fails to follow the treatment plan is not limited.

8 Observation and Assessment During Treatment

The following items need to be assessed during the treatment period:

a) Nasopharynx and neck MRI and nasopharyngeal endoscopy will be

performed before and after induce chemotherapy and at the end of radiotherapy, and complete response (CR), partial response (PR), stable disease (SD) or progressive disease (PD) will be evaluated with the Response Evaluation Criteria in Solid Tumors (RECIST) version 1.1. Chest CT and abdominal CT/MR will be reexamined after treatment.

b) General conditions.

c) Acute and late toxicity assessment, including hematological toxicity, gastrointestinal reactions, hepatotoxicity, nephrotoxicity, mucositis, neurotoxicity, ototoxicity, etc.

d) Immune-related toxicity assessment.

e) Laboratory tests: Routine blood tests and blood biochemistry are required on day 0, day 8, and day 15 of chemotherapy for each cycle; the following tests are routinely suggested but not mandated before each cycle of immunotherapy: thyroid function (TSH, FT3, FT4), coagulation function (APTT, PT, FIB, INR, D-D), serum amylase, lipase, myocardial enzyme profile, hypersensitivity troponin, brain natriuretic peptide, inflammatory cytokine profile, etc.

f) Indirect nasopharyngoscopy will be performed to examine the tumor in the nasopharynx every week, and the regression of enlarged lymph nodes should be observed and measured. Nasal endoscopy will be performed before and after the treatment course and is also required after each cycle of chemotherapy or immunotherapy.

9 Exploratory analysis and translational study

9.1 Exploratory analysis

Pathologic evaluation of endoscopic biopsy and/or lymph nodes biopsy after induction phase would be conducted. The primary efficacy endpoint was DMFS, which was defined as the interval between the initial treatment and distant failure.

EBV DNA clearance rate.

9.2 Translational study

a) Signed informed consent is required prior to each biological sample collection.

b) Tissue specimens from nasopharyngeal and/or neck mass biopsies or fine needle aspiration prior to treatment and after induction therapy, or from recurrence or metastasis site if recurrent or metastasis event occurred.

c) Blood samples, collected at the following time points: before treatment, after induction therapy, the end of radiotherapy, the end of maintain phase, and the time point of distant metastasis or recurrence events.

d) Clinical specimens for the above cases whose informed consent have been signed will be collected by designated professionals, and all specimens collected will be used for subject-related scientific research only.

e) After collection, tumor tissue specimens will be collected by the principal investigator and stored in a refrigerator at -80 °C or transferred to a designated laboratory for processing and analysis.

f) Hematology specimens are collected, centrifuged, and stored by the principal investigator, or transferred to the designated laboratory for biological sample processing and analysis.

10 Safety Evaluation

10.1 Adverse Event Indicators

Toxicity evaluation should be performed weekly during the treatment and four weeks after the end of treatment. All observations relevant to the safety of the drug under study will be documented in the case report form (CRF) and the final summary report. Safety indicators are as follows: adverse events, laboratory changes (hematology, blood

biochemistry), changes in vital signs (blood pressure, heart rate, respiratory rate, body temperature), and changes in electrocardiogram and chest CT.

10.2 Severe Adverse Events (SAEs)

A SAE is any life-threatening medical adverse event that occurs at a variety of drug doses:

a. Causing death or endangering life. It should be noted that the term "life-threatening" in the definition of "severe" means that at the time of the event, the patient is at risk of death. It's not a hypothetical event.
b. Causing permanent or severe disability/dysfunction.
c. Teratogenicity/birth defects.
d. Resulting in prolonged hospital stays.
e. Lead to secondary tumors.
f. Other unpredictable drug related AEs

In the event of a serious adverse event, regardless of whether it is related to the study drug, the investigator should immediately notify the Clinical Research Center (GCP) of the table of serious adverse events within 1 business day (in any case). The form must be dated and signed by the person who filled it

All adverse events, whether or not considered drug related, should be documented on the case report, including diagnosis, start/end time, intervention taken, discontinuation of treatment, salvage measure, outcome, and other possible causes. The investigators should determine the relationship of all adverse events to treatment and the severity of the events. The severity of acute adverse events should be graded according to CTC AE (version 5.0). The most severe severity should be recorded in the CRF.

10.3 Record of Events

All adverse events, whether or not considered drug related, should be documented on the case report, including diagnosis, start/end time, intervention taken,

discontinuation of treatment, salvage measure, outcome, and other possible causes. The investigators should determine the relationship of all adverse events to treatment and the severity of the events. The severity of acute adverse events should be graded according to CTC AE (version 5.0). The most severe severity should be recorded in the CRF.

11 Follow Up

After completion of radiotherapy, patients are followed up every 3 months during the first 3 years and every 6 months thereafter until death. The nasopharynx will be assessed by endoscopy approximately 4 weeks after completion of radiotherapy. Further investigations with MRI or CT will be arranged 3 months after completion of radiotherapy. Other follow-up items include physical examination, blood examination, chest CT, abdominal MR or CT, etc. Treatment responses are also evaluated according to the RECIST, version 1.1. If residual disease is found, whether to treat and which treatment modalities to be employed will be decided by individual clinician. For statistical purpose, any residual disease found 12 weeks after completion of RT will be considered as local failure. Similarly, any residual nodal disease within 12 weeks after RT is considered as regional failure.

Clinical diagnosis is accepted for sites not easily accessible if classical changes are shown on imaging. The dates of diagnosis of local, nodal, and distant failure will be recorded. The earliest date of detecting symptomatic late onset toxicities and the eventual maximum grade by the Late Radiation Morbidity Scoring Criteria of the Radiation Therapy Oncology Group (RTOG) and European Organization for Research and Treatment of Cancer (EORTC) should be recorded. All patients will be followed-up until death and cause of death recorded. Deaths due to unknown cause are counted as death due to NPC if disease is still present at last assessment.

12 Safety Measures and Quality Control

a) Researchers is responsible for obtaining informed consent signed by each subject or his or her agent; carefully fill out the case report form; complete records of laboratory examinations, clinical records, and original medical records of the subjects.

b) Make monitoring plan of adverse effects and emergency plan.

c) Research plan is made by all investigators and approved by Ethics Committee.

d) Develop all kinds of standard operation procedures related to this study.

e) Establish standardized evaluation system to unify diagnostic criteria, curative effect judging criteria, etc.

f) Establish professional statistical plan.

g) Research staffs are trained before the study.

h) Arrange quality controller, make quality control plan and check regularly.

i) Set up coordination committee, curative effect judging group and follow-up team.

13 Data Collection and Data Management

13.1 Case Report Form (CRF)

The CRF is completed by the investigator and must be completed by each enrolled patient. After the completed CRF is reviewed by the clinical supervisor, the first copy is transferred to the data administrator for data entry and management.

13.2 Data Entry and Modification

All information about the enrolled patients after registration will be sent to Sun Yat-Sen University Cancer Center for management. We have stewards taking charge of data entry and management. Data administrators use EpiData 3.1 software to compile

data entry program and carry out data entry and management. In order to ensure the accuracy of the data, two data administrators should independently conduct double input and proofreading. If there are any questions in the CRF, the data administrator will send questioned data to the investigator for verification. The investigator should answer and return the questions as soon as possible. The data administrator can modify, confirm and enter the data according to the answers of the investigator.

13.3 Data Locking

After confirming that the established database is accurate, the main researchers, data administrators, and statisticians will lock the data. The locked data file will not be modified.

14 Statistical Analysis

14.1 Endpoint Definitions

14.1.3 Primary End Point

The primary endpoint was DMFS, which was defined as the time from the initiation of treatment to documented distant metastasis.

14.1.4 Secondary End Points

Response rate. Approximately one week after completing induction therapy, the responses to induction therapy are assessed by imaging by independent image committee. The nasopharynx and cervical nodes should be assessed by endoscopy and MRI or CT at approximately 12 weeks after completion of radiotherapy. Other examinations included chest CT, abdominal CT/MR, and bone scans. Tumor response is classified according to the RECIST criteria, version 1.1. Complete response is

defined as the disappearance of all target lesions. Any pathological lymph nodes (whether target or nontarget) must have been reduced in the short axis to <10 mm. Partial response is defined as an at least 30% decrease in the sum of diameters of the target lesions, with the baseline diameter sum serving as the reference. Progressive disease is defined as an at least 20% increase in the sum of diameters of the target lesions, with the smallest sum during study serving as the reference (including the baseline sum). In addition to a relative increase of 20%, the sum must also demonstrate an absolute increase of at least 5 mm. Stable disease is defined as both insufficient size reduction to qualify as partial response and an insufficient increase to be considered progressive disease, with the smallest diameter sum during the study serving as the reference.

PFS, which is defined as the time from the initiation of induction therapy to documented distant metastasis, locoregional recurrence, or death from any cause, whichever occurred first

OS, which is defined as the time from the initiation of induction therapy to any-cause death.

LRFS, which is defined as the time from the initiation of induction therapy to documented locoregional recurrence.

Treatment toxicity. The incidence of serious toxicity (treatment-related adverse events and immune-related adverse events) during induction phase, radiotherapy phase, and maintain phase, late adverse events, compliance to treatment were included. Adverse events refer to any adverse medical events that occur on the patient. They do not necessarily have a causal relationship with treatment. Investigators should keep a detailed record of any adverse events that occur in the patients. The record of adverse events shall include a description of the adverse events, the time of occurrence, severity, duration, measures taken, and the final outcomes. Investigators should assess the possible association between the adverse events and the tested drugs according to the five-level classification of "positive relevance, possible irrelevance, positive irrelevance, and inability to determine." Treatment-related adverse events are assessed according to NCI-CTCAE version 5.0. Acute toxicities include hematological toxicity,

mucositis, allergic reactions and other adverse events and serious adverse events. The severity of immune-related adverse events was graded according to the NCI-CTCAE version 5.0. The severity of late adverse events was graded according to the Radiation Therapy Oncology Group and European Organization for Research and Treatment of Cancer late radiation morbidity scoring scheme.

Quality of life. EORTC QLQ-C30 and QLQ-H&N35 (v1.0) are used to assess life quality of patients, and the change of their life quality is recorded and evaluated weekly from before the beginning of treatment to the last dose of the camrelizumab.

14.2 Determination of the Sample Size

Considering over half distant metastasis events occurred within one year after radical treatment, the primary endpoint of this study was the DMFS at the time point of 1 year. The following set of hypotheses were considered:

H_0: The maximum 1-year DMFS rate of treatment cohort is not more than 76%;

H_A: The minimum 1-year DMFS rate of treatment cohort is not less than 90%.

The historical data estimation is based on the prognostic data of N3 population reported from our center.[13] According to the Fleming single-stage design, the maximum effective rate (P0) was 76%, the minimum effective rate (P1) was 90%, unilateral α was set as 0.05, and the power was set at least 80%, 46 patients should be enrolled. Assuming an 8% dropout rate, we estimated that the study would need to include a total of 50 patients. If 7 of the first 46 evaluable patients failed, the DMFS rate would be considered unacceptable, and the regimen would be declared promising if at least 40 successes of the first 46 evaluable patients were observed.

14.3 Analytical Approach

The therapeutic effects will be analyzed by both full analysis set (intention-to-treat) and per-protocol set. Patients who completed radical radiotherapy and at least one dose of induction therapy were included in the per-protocol analysis set. Safety analysis will be performed in safety population, which is made up of eligible cases that received at

least one dose of protocol-defined treatment.

The study results will be reported after universal 1-year follow-up (for the primary endpoint analysis), 2-year and/or 3-year follow-up, and 5-year follow-up (for the long-term results).

15 Ethical Considerations

This study must be approved by an appropriate institutional ethic committee.

An informed consent must be obtained from individual patients. Copy of the Consent Form, contact number of investigator and ethics committee will be available to patient on request.

All serious and unexpected adverse events or death related to the drugs or radiotherapy must be reported to the study coordinator immediately. Serious adverse events (SAE) to be reported include all deaths during or within 30 days of protocol treatment regardless of cause, grade 5 toxicity, life-threatening grade 4 toxicity, and/or unexpected toxicity. The Study Coordinator of respective center should complete form and fax this within 24 hours to the Principal Investigator (Dr. Yan-Qun Xiang, Tel: 020-87343379, Fax: 020-87343379), the center of clinical trials, the institutional ethic committee and Sun Yat-Sen University Cancer Center. Together with the Principal Investigator, appropriate and prompt action will be taken if warranted. Reactions and deaths beyond 30 days from protocol treatment that are judged definitely unrelated to treatment should not be reported.

16 Administration of Experimental Drugs

Jiangsu Hengrui Pharmaceutical provided nab-paclitaxel and apatinib free of charge and camrelizumab free of partial charge. The management, distribution, and recovery of clinical drugs in this trial shall be the responsibility of designated researcher.

The researcher must ensure that all trial drugs are used only for subjects participating in the clinical trial, that their doses and usage are in accordance with the trial scheme, and that the remaining drugs are returned to the manufacturer. Experimental drugs should not be transferred to any non-clinical trial participant.

17 Progress of Study

Expected trial start time: May 2020;

Expected completion time: May 2021;

Expected end of trial: May 2026.

18 Principle center and investigator

Principle center: Sun Yat-Sen University Cancer Center

Principle Investigator: Yan-Qun Xiang

Address: Dongfeng East Road 651, Guangzhou 510060

Telephone: 020-87343379

Fax: 020-87343379

19 Bibliography

1. Chen YP, Chan ATC, Le QT, et al: Nasopharyngeal carcinoma. Lancet 394:64-80, 2019
2. Huang ZL, Liu S, Wang GN, et al: The prognostic significance of PD-L1 and PD-1 expression in patients with nasopharyngeal carcinoma: a systematic review and meta-analysis. Cancer Cell Int 19:141, 2019
3. Mai HQ, Chen QY, Chen D, et al: Toripalimab or placebo plus chemotherapy as first-line treatment in advanced nasopharyngeal carcinoma: a multicenter randomized phase 3 trial. Nat Med 27:1536-1543, 2021
4. Wang FH, Wei XL, Feng J, et al: Efficacy, Safety, and Correlative Biomarkers of Toripalimab in Previously Treated Recurrent or Metastatic Nasopharyngeal Carcinoma: A Phase II Clinical Trial (POLARIS-02). J Clin Oncol 39:704-712, 2021
5. Fang W, Yang Y, Ma Y, et al: Camrelizumab (SHR-1210) alone or in combination with gemcitabine plus cisplatin for nasopharyngeal carcinoma: results from two single-arm, phase 1 trials. Lancet Oncol 19:1338-1350, 2018
6. Yang Y, Qu S, Li J, et al: Camrelizumab versus placebo in combination with gemcitabine and cisplatin as first-line treatment for recurrent or metastatic nasopharyngeal carcinoma (CAPTAIN-1st): a multicentre, randomised, double-blind, phase 3 trial. Lancet Oncol 22:1162-1174, 2021
7. Schmittnaegel M, Rigamonti N, Kadioglu E, et al: Dual angiopoietin-2 and VEGFA inhibition elicits antitumor immunity that is enhanced by PD-1 checkpoint blockade. Sci Transl Med 9, 2017
8. Allen E, Jabouille A, Rivera LB, et al: Combined antiangiogenic and anti-PD-L1 therapy stimulates tumor immunity through HEV formation. Sci Transl Med 9, 2017
9. Xu J, Zhang Y, Jia R, et al: Anti-PD-1 Antibody SHR-1210 Combined with Apatinib for Advanced Hepatocellular Carcinoma, Gastric, or Esophagogastric Junction Cancer: An Open-label, Dose Escalation and Expansion Study. Clin Cancer Res 25:515-523, 2019
10. Li Q, Wang Y, Jia W, et al: Low-Dose Anti-Angiogenic Therapy Sensitizes Breast Cancer to PD-1 Blockade. Clin Cancer Res 26:1712-1724, 2020
11. Liu J, Liu Q, Li Y, et al: Efficacy and safety of camrelizumab combined with apatinib in advanced triple-negative breast cancer: an open-label phase II trial. J Immunother Cancer 8, 2020
12. Xie L, Xu J, Sun X, et al: Apatinib plus camrelizumab (anti-PD1 therapy, SHR-1210) for advanced osteosarcoma (APFAO) progressing after chemotherapy: a single-arm, open-label, phase 2 trial. J Immunother Cancer 8, 2020
13. Sun X, Su S, Chen C, et al: Long-term outcomes of intensity-modulated radiotherapy for 868 patients with nasopharyngeal carcinoma: an analysis of survival and treatment toxicities. Radiother Oncol 110:398-403, 2014

Appendix I: The 8th editions of the AJCC Staging System of Nasopharyngeal Carcinoma

**Tumor stage**	
Tx	Primary tumour cannot be assessed
T0	No tumour identified, but there is EBV-positive cervical node(s) involvement
T1	Nasopharynx, oropharynx, or nasal cavity without parapharyngeal extension
T2	Parapharyngeal extension, adjacent soft tissue involvement (medial pterygoid, lateral pterygoid, prevertebral muscles)
T3	Bony structures (skull base, cervical vertebra) and/or paranasal sinuses
T4	Intracranial extension, cranial nerve, hypopharynx, orbit, extensive soft tissue involvement (beyond the lateral surface of the lateral pterygoid muscle, parotid gland)
**Node stage**	
Nx	Regional lymph nodes cannot be assessed
N0	No regional lymph node metastasis
N1	Unilateral cervical, unilateral, or bilateral retropharyngeal lymph nodes, above the caudal border of cricoid cartilage; ≤ 6 cm
N2	Bilateral metastasis in lymph node(s), ≤ 6 cm in greatest dimension, above the caudal border of cricoid cartilage
N3	> 6 cm and/or below caudal border of cricoid cartilage (regardless of laterality)
**Metastasis**	
M0	No distant metastasis
M1	Distant metastasis
**TNM stage**	
I	T1 N0 M0
II	T2 N0–1 M0, T0–1 N1 M0
III	T3 N0–2 M0, T0–2 N2 M0
IVa	T4 or N3 M0
IVb	Any T, any N, M1

Appendix II: ECOG Performance Status and Scores

Score	ECOG Performance status*
0	Fully active, able to carry on all pre-disease performance without restriction
1	Restricted in physically strenuous activity but ambulatory and able to carry out work of a light or sedentary nature, e.g., light housework, office work
2	Ambulatory and capable of all selfcare but unable to carry out any work activities; up and about more than 50% of waking hours
3	Capable of only limited selfcare; confined to bed or chair more than 50% of waking hours
4	Completely disabled; cannot carry on any selfcare; totally confined to bed or chair
5	Dead
* Oken M, Creech R, Tormey D, et al. Toxicity and response criteria of the Eastern Cooperative Oncology Group. Am J Clin Oncol. 1982;5:649-655.<br>Note: ECOG denotes Eastern Cooperative Oncology Group	

Appendix III: Acute Induction Chemotherapy and Chemoradiotherapy Toxicity Graded by Common Terminology Criteria for Adverse Events (CTCAE 5.0).

The CTCAE v5.0 manual can be found at the following URL: https://evs.nci.nih.gov/ftp1/CTCAE/CTCAE_5.0/

Appendix IV: Late Onset Toxicities Graded by RTOG/EORTC System

RTOG/EORTC System manual can be found at the following URL: https://www.eortc.org/rog-guidelines/

Summary of changes in protocol

Page	Summary	Changes
56	We added detailed information of the usage of camrelizumab.	In particular, an actual dose of 3 mg/kg was given to those weighing less than 40 kg.
56	Based on the tolerability of the enrolled patients, we modified the initial dosage of apatinib.	Apatinib taken orally at a dose of 250 mg daily on days 1 to 5 per week for a total of 8 weeks.
66	Due to the adjustment initial dosage of apatinib, we deleted description of the first adjustment dosage correspondingly.	~~250 mg daily, 5 days every week for the first adjustment;~~
67	We added detailed information of the dosage adjustment of apatinib.	For patients with symptoms of any-reason nosebleed, apatinib should be suspended until the bleeding disappears and apatinib can be continued without dose adjustment.
70	We added detailed information of the dosage adjustment of camrelizumab.	For patients with reactive capillary endothelial proliferation in mucous membrane-related sites such as the oral cavity or nasal cavity that cause bleeding symptoms, symptomatic support should be provided and camrelizumab should be suspended and resumed when the aforementioned reactive capillary endothelial proliferation in mucous membrane sites has resolved or when no risk of potential major bleeding (>100 ml) has been assessed by the investigators.
70	We added detailed information of the dosage adjustment of camrelizumab.	Considering the diversity of causes of rash reactions during combination drug administration, the established treatment regimen was maintained for patients who experienced grade 4 rash reactions that subsided after one week using symptomatic treatment. Patients whose rash reactions persisted for more than two weeks without complete remission, camrelizumab was suspended and chemotherapy drugs continued; and a second cautious attempt may be considered after complete resolution of symptoms evaluated by the investigators and after full communication with the patient.

Original statistical analysis plan

1 Statistical Analysis

1.1 Endpoint Definitions

1.1.1 Primary End Point

The primary endpoint was DMFS, which was defined as the time from the initiation of treatment to documented distant metastasis.

1.1.2 Secondary End Points

Response rate. Approximately one week after completing induction therapy, the responses to induction therapy are assessed by imaging by independent image committee. The nasopharynx and cervical nodes should be assessed by endoscopy and MRI or CT at approximately 12 weeks after completion of radiotherapy. Other examinations included chest CT, abdominal CT/MR, and bone scans. Tumor response is classified according to the RECIST criteria, version 1.1. Complete response is defined as the disappearance of all target lesions. Any pathological lymph nodes (whether target or nontarget) must have been reduced in the short axis to <10 mm. Partial response is defined as an at least 30% decrease in the sum of diameters of the target lesions, with the baseline diameter sum serving as the reference. Progressive disease is defined as an at least 20% increase in the sum of diameters of the target lesions, with the smallest sum during study serving as the reference (including the baseline sum). In addition to a relative increase of 20%, the sum must also demonstrate an absolute increase of at least 5 mm. Stable disease is defined as both insufficient size reduction to qualify as partial response and an insufficient increase to be considered progressive disease, with the smallest diameter sum during the study serving as the reference.

FFS, which is defined as the time from the initiation of induction therapy to documented distant metastasis, locoregional recurrence, or death from any cause, whichever occurred first

OS, which is defined as the time from the initiation of induction therapy to any-

cause death.

LRFS, which is defined as the time from the initiation of induction therapy to documented locoregional recurrence.

Treatment toxicity. The incidence of serious toxicity (treatment-related adverse events and immune-related adverse events) during induction phase, radiotherapy phase, and maintain phase, late adverse events, compliance to treatment were included. Adverse events refer to any adverse medical events that occur on the patient. They do not necessarily have a causal relationship with treatment. Investigators should keep a detailed record of any adverse events that occur in the patients. The record of adverse events shall include a description of the adverse events, the time of occurrence, severity, duration, measures taken, and the final outcomes. Investigators should assess the possible association between the adverse events and the tested drugs according to the five-level classification of "positive relevance, possible irrelevance, positive irrelevance, and inability to determine." Treatment-related adverse events are assessed according to NCI-CTCAE version 5.0. Acute toxicities include hematological toxicity, mucositis, allergic reactions and other adverse events and serious adverse events. The severity of immune-related adverse events was graded according to the NCI-CTCAE version 5.0. The severity of late adverse events was graded according to the Radiation Therapy Oncology Group and European Organization for Research and Treatment of Cancer late radiation morbidity scoring scheme.

Quality of life. EORTC QLQ-C30 and QLQ-H&N35 (v1.0) are used to assess life quality of patients, and the change of their life quality is recorded and evaluated weekly from before the beginning of treatment to the last dose of the camrelizumab.

1.2 Determination of the Sample Size

Considering over half distant metastasis events occurred within one year after radical treatment, the primary endpoint of this study was the DMFS at the time point of 1 year. The following set of hypotheses were considered:

H0: The maximum 1-year DMFS rate of treatment cohort is not more than 76%;

HA: The minimum 1-year DMFS rate of treatment cohort is not less than 90%.

The historical data estimation is based on the prognostic data of N3 population

reported from our center.13 According to the Fleming single-stage design, the maximum effective rate (P0) was 76%, the minimum effective rate (P1) was 90%, unilateral α was set as 0.05, and the power was set at least 80%, 46 patients should be enrolled. Assuming an 8% dropout rate, we estimated that the study would need to include a total of 50 patients. If 7 of the first 46 evaluable patients failed, the DMFS rate would be considered unacceptable, and the regimen would be declared promising if at least 40 successes of the first 46 evaluable patients were observed.

1.3 Analytical Approach

The therapeutic effects will be analyzed by both full analysis set (intention-to-treat) and per-protocol set. Patients who completed radical radiotherapy and at least one dose of induction therapy were included in the per-protocol analysis set. Safety analysis will be performed in safety population, which is made up of eligible cases that received at least one dose of protocol-defined treatment.

The study results will be reported after universal 1-year follow-up (for the primary endpoint analysis), 2-year and/or 3-year follow-up, and 5-year follow-up (for the long-term results).
