# Supplementary material for: Camrelizumab and apatinib plus induction chemotherapy and concurrent chemoradiotherapy in stage N3 nasopharyngeal carcinoma: a phase 2 clinical trial
Source: Nat Commun. 2024 Feb 3;15:1029. doi: 10.1038/s41467-024-45126-0 (PMC10838332; doi:10.1038/s41467-024-45126-0)
Supplement: Supplementary file 3 — Reporting Summary [file 41467_2024_45126_MOESM3_ESM.pdf]

nature portfolio

Corresponding author(s): Yan-Qun Xiang

Last updated by author(s): Jan 16, 2024

Reporting Summary

Nature Portfolio wishes to improve the reproducibility of the work that we publish. This form provides structure for consistency and transparency in reporting. For further information on Nature Portfolio policies, see our Editorial Policies and the Editorial Policy Checklist.

Statistics

For all statistical analyses, confirm that the following items are present in the figure legend, table legend, main text, or Methods section.

n/a	Confirmed	
☐	☒	The exact sample size (*n*) for each experimental group/condition, given as a discrete number and unit of measurement
☒	☐	A statement on whether measurements were taken from distinct samples or whether the same sample was measured repeatedly
☐	☒	The statistical test(s) used AND whether they are one- or two-sided<br>*Only common tests should be described solely by name; describe more complex techniques in the Methods section.*
☒	☐	A description of all covariates tested
☒	☐	A description of any assumptions or corrections, such as tests of normality and adjustment for multiple comparisons
☐	☒	A full description of the statistical parameters including central tendency (e.g. means) or other basic estimates (e.g. regression coefficient) AND variation (e.g. standard deviation) or associated estimates of uncertainty (e.g. confidence intervals)
☐	☒	For null hypothesis testing, the test statistic (e.g. *F*, *t*, *r*) with confidence intervals, effect sizes, degrees of freedom and *P* value noted<br>*Give P values as exact values whenever suitable.*
☒	☐	For Bayesian analysis, information on the choice of priors and Markov chain Monte Carlo settings
☒	☐	For hierarchical and complex designs, identification of the appropriate level for tests and full reporting of outcomes
☒	☐	Estimates of effect sizes (e.g. Cohen's *d*, Pearson's *r*), indicating how they were calculated

Our web collection on statistics for biologists contains articles on many of the points above.

Software and code

Policy information about availability of computer code

Data collection	The data in this study were collected using the EpiData 3.1.
Data analysis	The data in this study were analyzed using the Stata software, version 14.2 (StataCorp).

For manuscripts utilizing custom algorithms or software that are central to the research but not yet described in published literature, software must be made available to editors and reviewers. We strongly encourage code deposition in a community repository (e.g. GitHub). See the Nature Portfolio guidelines for submitting code & software for further information.

Data

Policy information about availability of data

All manuscripts must include a data availability statement. This statement should provide the following information, where applicable:

- Accession codes, unique identifiers, or web links for publicly available datasets
- A description of any restrictions on data availability
- For clinical datasets or third party data, please ensure that the statement adheres to our policy

Source data are not publicly available due to involving patient privacy, but can be accessed on request from the corresponding author for 10 years. Any request should be sent to the corresponding author, Yan-Qun Xiang, via email at xiangyq@sysucc.org.cn, along with a detailed description of your research protocol; individual deidentified participant data will be shared. The corresponding author and Sun Yat-sen University Cancer Center will evaluate the reasonability of the request for our data and reserve the right to decide whether to share the data or not. And the data is only used for the research purpose. This process requires an average of 6 months. All data shared will be de-identified and will be available for 1 year after access is granted. The study protocol is available as Supplementary

Note in the Supplementary Information file. The remaining data are available within the Article, Supplementary Information.

Research involving human participants, their data, or biological material

Policy information about studies with human participants or human data. See also policy information about sex, gender (identity/presentation), and sexual orientation and race, ethnicity and racism.

Reporting on sex and gender	Sex was self-report and was not considered in study design. In the Table 1, gender analysis was performed.37 patients are male and 12 patients are female in the study.
Reporting on race, ethnicity, or other socially relevant groupings	The manuscript used gender and age as the socially constructed or socially relevant categorization variables.The study aimed to examine the effect of gender and age on ORR and survival in different cohorts.Gender was self-reported by participants as male or female. Age was also self-reported as a continuous variable in years.There was no confounding variables in our analyses.The study did not onfounding variables, such as education, income, political ideology, and geographic region.
Population characteristics	Table 1 lists baseline characteristics of the population.
Recruitment	Participants were recruited from the Guangzhou area (China) via posters, emails, flyers, social media, and website advertisements. 49 participants were recruited into the trial after signing a written informed consent. Because all the participants were recruited from the Guangzhou area, they might not have been representative of the wider population. No other potential self-selection bias was present in this trial.
Ethics oversight	The trial protocol was approved by the Chinese Ethics Committee of Registering Clinical Trials.All patients provided written informed consent.

Note that full information on the approval of the study protocol must also be provided in the manuscript.

Field-specific reporting

Please select the one below that is the best fit for your research. If you are not sure, read the appropriate sections before making your selection.

- [x] Life sciences
- [ ] Behavioural & social sciences
- [ ] Ecological, evolutionary & environmental sciences

For a reference copy of the document with all sections, see nature.com/documents/nr-reporting-summary-flat.pdf

Life sciences study design

All studies must disclose on these points even when the disclosure is negative.

Sample size	Considering over half distant metastasis events occurred within one year after radical treatment, the primary endpoint of this study was the DMFS at the time point of 1 year. The following set of hypotheses were considered:<br>H0: The maximum 1-year DMFS rate of treatment cohort is not more than 76%;<br>HA: The minimum 1-year DMFS rate of treatment cohort is not less than 90%.<br>The historical data estimation is based on the prognostic data of N3 population reported from our center. According to the Fleming single-stage design, the maximum effective rate (P0) was 76%, the minimum effective rate (P1) was 90%, unilateral α was set as 0.05, and the power was set at least 80%, 46 patients should be enrolled. Assuming an 8% dropout rate, we estimated that the study would need to include a total of 50 patients. If 7 of the first 46 evaluable patients failed, the DMFS rate would be considered unacceptable, and the regimen would be declared promising if at least 40 successes of the first 46 evaluable patients were observed.
Data exclusions	A history of previous radiotherapy, chemotherapy, or surgery to the primary tumor or lymph nodes; any severe coexisting diseases; treatment with palliative intention; previous malignancy; pregnancy or lactation; a history of protocol-specified autoimmune disease.
Replication	No measures taken to verify the reproducibility of the experimental findings.
Randomization	This is a single arm phase 2 study.
Blinding	This is a Phase II small sample study, so the treatment of all patients is consistent.

Reporting for specific materials, systems and methods

We require information from authors about some types of materials, experimental systems and methods used in many studies. Here, indicate whether each material, system or method listed is relevant to your study. If you are not sure if a list item applies to your research, read the appropriate section before selecting a response.

Materials & experimental systems

n/a	Involved in the study
☒	☐ Antibodies
☒	☐ Eukaryotic cell lines
☒	☐ Palaeontology and archaeology
☒	☐ Animals and other organisms
☐	☒ Clinical data
☒	☐ Dual use research of concern
☒	☐ Plants

Methods

n/a	Involved in the study
☒	☐ ChIP-seq
☒	☐ Flow cytometry
☒	☐ MRI-based neuroimaging

Clinical data

Policy information about clinical studies
All manuscripts should comply with the ICMJEguidelines for publication of clinical research and a completedCONSORT checklist must be included with all submissions.

Clinical trial registration: ChiCTR2000032317

Study protocol: Supplementary protocol

Data collection: From May 2020 to July 2021, stage TanyN3M0 NPC were treated at Sun Yat-Sen University Cancer Center

Outcomes: The primary endpoint was DMFS, defined as the time from the initiation of induction therapy to the first documented distant metastasis. Secondary endpoints were overall survival (OS), FFS, locoregional recurrence-free survival (LRFS), tumor response, and toxicity profile. FFS, which is defined as the time from the initiation of induction therapy to documented distant metastasis, locoregional recurrence, or death from any cause, whichever occurred first.

OS, which is defined as the time from the initiation of induction therapy to any-cause death.

LRFS, which is defined as the time from the initiation of induction therapy to documented locoregional recurrence.

Treatment toxicity. The incidence of serious toxicity (treatment-related adverse events and immune-related adverse events) during induction phase, radiotherapy phase, and maintain phase, late adverse events, compliance to treatment were included. Adverse events refer to any adverse medical events that occur on the patient. They do not necessarily have a causal relationship with treatment. Investigators should keep a detailed record of any adverse events that occur in the patients. The record of adverse events shall include a description of the adverse events, the time of occurrence, severity, duration, measures taken, and the final outcomes. Investigators should assess the possible association between the adverse events and the tested drugs according to the five-level classification of "positive relevance, possible irrelevance, positive irrelevance, and inability to determine." Treatment-related adverse events are assessed according to NCI-CTCAE version 5.0. Acute toxicities include hematological toxicity, mucositis, allergic reactions and other adverse events and serious adverse events. The severity of immune-related adverse events was graded according to the NCI-CTCAE version 5.0. The severity of late adverse events was graded according to the Radiation Therapy Oncology Group and European Organization for Research and Treatment of Cancer late radiation morbidity scoring scheme.

Plants

Seed stocks: NA

Novel plant genotypes: NA

Authentication: NA
